# The Psychonauts’ Benzodiazepines; Quantitative Structure-Activity Relationship (QSAR) Analysis and Docking Prediction of Their Biological Activity

**DOI:** 10.3390/ph14080720

**Published:** 2021-07-26

**Authors:** Valeria Catalani, Michelle Botha, John Martin Corkery, Amira Guirguis, Alessandro Vento, Norbert Scherbaum, Fabrizio Schifano

**Affiliations:** 1 Psychopharmacology, Drug Misuse & Novel Psychoactive Substances Research Unit, School of Life & Medical Sciences, University of Hertfordshire, College Lane Campus, Hatfield AL10 9AB, UK; v.catalani@herts.ac.uk (V.C.); m.botha@herts.ac.uk (M.B.); j.corkery@herts.ac.uk (J.M.C.); amira.guirguis@swansea.ac.uk (A.G.); f.schifano@herts.ac.uk (F.S.); 2 Department of Pharmacy, Swansea University Medical School, Faculty of Medicine, Health and Life Science, Swansea University, Singleton Campus, Wales SA2 8PP, UK; 3 Department of Mental Health, ASL Roma 2, 00182 Rome, Italy; alessandrovento@gmail.com; 4 Addictions’ Observatory (ODDPSS), 00141 Rome, Italy; 5 Department of Psychology, Guglielmo Marconi University, 00193 Rome, Italy; 6 LVR-Hospital Essen, Department of Psychiatry and Psychotherapy, Medical Faculty, University of Duisburg-Essen, 45147 Essen, Germany

**Keywords:** designer benzodiazepines, QSAR, docking, web crawler, computational models, MOE, NPS*finder*^®^

## Abstract

Designer benzodiazepines (DBZDs) represent a serious health concern and are increasingly reported in polydrug consumption-related fatalities. When new DBZDs are identified, very limited information is available on their pharmacodynamics. Here, computational models (i.e., quantitative structure-activity relationship/QSAR and Molecular Docking) were used to analyse DBZDs identified online by an automated web crawler (NPS*finder*^®^) and to predict their possible activity/affinity on the gamma-aminobutyric acid A receptors (GABA-ARs). The computational software MOE was used to calculate 2D QSAR models, perform docking studies on crystallised GABA-A receptors (6HUO, 6HUP) and generate pharmacophore queries from the docking conformational results. 101 DBZDs were identified online by NPS*finder*^®^. The validated QSAR model predicted high biological activity values for 41% of these DBDZs. These predictions were supported by the docking studies (good binding affinity) and the pharmacophore modelling confirmed the importance of the presence and location of hydrophobic and polar functions identified by QSAR. This study confirms once again the importance of web-based analysis in the assessment of drug scenarios (DBZDs), and how computational models could be used to acquire fast and reliable information on biological activity for index novel DBZDs, as preliminary data for further investigations.

## 1. Introduction

Benzodiazepines (BZDs) were introduced into therapeutics in the early 1960s as a safer alternative to barbiturates, but their abuse potential was recognised early on [1]. For this reason, 35 BZDs were placed under control by the UN Convention on Psychotropic Substances of 1971 [2,3]. Both their relatively low toxicity levels and their activity as anxiolytics, sedative-hypnotics, anticonvulsants, and muscle relaxants made and still make them one of the most prescribed classes of drugs across the world [4,5].

The BDZ core structure is a (1,4)-diazepine fused to a benzene ring. A phenyl moiety is usually attached to this core. They act as positive allosteric modulators of γ-aminobutyric acid (GABA)-A receptor (GABA-AR) [6]. The GABA-AR is a heteropentameric unit (i.e., 5 glycoproteins: 2 α, 2 β and 1 γ s). The α, β and γ subunits show different isoforms to which correspond a diverse activity of BZDs [7,8]. The α1 receptors are responsible for sedative, anterograde amnesic, and anticonvulsant actions; the α2 for anxiolytic effect and the α2,3,5 for myorelaxant actions [9,10]. The α1 isoform has been proven as being responsible for the addictive potential of BDZs [9,11].

BZDs have been long associated with a history of abuse, illicit trading and non-medical use, the latter being a well-established problem associated with many overdose-related deaths worldwide, notwithstanding the availability of flumazenil, the BZD antagonist, in specialized settings [12,13]. The adverse effects associated with their use/abuse include increased reaction time, motor incoordination, anterograde amnesia, restlessness, delirium, aggression, depression, hallucinations, paranoia and fatalities [14].

The health concern associated with these molecules has increased recently due to the appearance on the market of several new BZD analogues, belonging to the group of new psychoactive substances (NPS) [15,16,17]. Even though the family of designer BZDs (DBZDs) represent less than 2% of the total available NPSs [18,19], in the last couple of years, they have been reported worldwide in most NPS hospitalisations and fatalities (post-mortem) [20], and particularly so in polydrug consumption scenarios [21,22,23,24]. They are usually ingested with other sedatives, primarily opioids, to potentiate their effects; promote ‘come down’ after stimulant use; or unintentionally, as counterfeits of prescription benzodiazepines [20]. Safety/toxicity profiles for most of the known DBZDs are not described yet, with limited knowledge of their pharmacodynamics and pharmacokinetics [15,25,26]. To date, the consumption of these new and unknown molecules is regarded as a serious threat to public health and society [20,27,28].

Currently, the United Nations Office on Drugs and Crime (UNODC) and the European Monitoring Centre for Drugs and Drugs Addiction (EMCDDA) report a total of 30 officially identified DBZDs [5], 70% of which were identified since 2015. However, results from previous studies conducted on the open web [29,30,31,32] demonstrate that the number of NPS, including DBZDs, identified online is much larger than that formally identified by either the EMCDDA and/or the UNODC databases [33,34]. Indeed, web-based studies have demonstrated their ability in both assessing and somewhat predicting the real-world scenario of NPS availability [35,36,37,38]. Web-based studies typically focus on activities carried out within both online drug forum communities and social networks, where there are some educated/informed users (‘psychonauts’) [39,40] who are keen to ‘test’ a range of molecules to achieve specific mindsets. Their information is routinely shared online, and vulnerable subjects, including both children/adolescents and psychiatric patients, may hence be at risk of accessing these ‘pro-drug’ data [39,40]. Psychonauts are a clinical ‘niche’, and their idiosyncratic levels of medication intake are better scrutinized with the help of the netnographic research approach [41]. To facilitate identification of those NPS being discussed and offered for sale on the surface web, a crawling/navigating software (i.e., NPS*finder*^®^), designed by Damicom, an IT enterprise based in Rome (Italy), was used. The crawler was designed to automatically scan a list of websites for new/novel/emerging NPS and extract a range of information including chemical and street names; chemical formulae; three-dimensional images; and anecdotally reported clinical/psychoactive effects. The scanned websites (Table S1) were representative of online psychonaut websites/fora and other NPS online resources, e.g., vendor websites [42].

However, when new NPS, including DBZDs, are identified online, only very limited, if any at all, scientific information is available on their activity/toxicity and carrying out preclinical studies on dozens, or hundreds, of molecules may constitute an extremely time-consuming and very expensive exercise [43]. Hence, to overcome this issue, computational models (e.g., quantitative structure-activity relationship/QSAR and Molecular Docking) have been suggested to help in providing levels of prediction of both biological activity and binding affinity of unknown molecules towards a known receptor. Indeed, computational models are well established, and successfully/extensively used tools, and especially so for drug development [44]. They have already been applied to classical and designer benzodiazepines [45,46] and other NPS classes (e.g., synthetic cannabinoids [47,48,49], opioids [50,51], hallucinogenic phenylalkylamines [52], and tryptamines [53] and phenethylamines [54,55].

This study aimed to use computational models to analyse the DBZDs identified online with the use of an automated web crawler (i.e., NPS*finder*^®^) and to predict their possible activity and potency on the gamma-aminobutyric acid receptors (GABA-ARs).

## 2. Results

The web crawler identified online a total of 4334 NPS [56]. After careful manual analysis and evaluation, 101 molecules among these NPSs were classified as DBDZs. These included all the molecules being formally listed and officially reported by the UNODC and EMCDDA (Table S2). For the scope of this paper, the controlled BDZs being listed in the Convention on Psychotropic Substances of 1971 were excluded, as they did not qualify as designer benzodiazepines.

### 2.1. QSAR

The dataset obtained from the literature consisted of 76 molecules, and included 1,4-benzodiazepines, thienotriazolo-benzodiazepines, triazolo-benzodiazepines, and imidazo-benzodiazepines [57]. The 76 molecules were divided further into a training (67) and a test set (9) and a SMILES string was generated for each molecule (Table S3). As per the methods section, the training set and test set composition was determined by similarity and activity sampling [58]. All the molecules presented experimental values of log1/c between 6.0 and 9.0, where the highest values correspond to higher biological activity.

The AutoQSAR application in MOE generated 80 QSAR models from the 260 2D descriptors calculated. To improve the results, the function “ignore outliers” was used. Only one compound (Ro 06-9098) was flagged by the AutoQSAR application as an outlier and not used for the model building. The QSAR equations (80) were manually filtered, aiming for a model with high values (>0.7) of r^2^ (goodness-to-fit), high values (>0.6) for xr^2^ (robustness) and fewer descriptors. The model that best represented this compromise was a 5-descriptors equation with an r^2^ of 0.75 and an xr^2^ of 0.69 (Equation (1)). Once chosen, the model was then applied on the test set for the external validation, obtaining an r^2^ = 0.66 as a measure of predictiveness and an RMSE value of 0.65. The r^2^ for the test set was calculated with the use of the correlation plot function in MOE. The predicted log1/c values for the test set were calculated with the “Model-Evaluate” function and then plotted against the experimental values with the “Analysis-Correlation plot”, which returned the r^2^ value. A better result (RMSE = 0.36) was achieved when two outliers (Ro 05-4336, Ro 05-2921) were removed from the validation set [59] as suggested in the literature.

Equation (1) QSAR Equation identified with AutoQSAR MOE application.

log 1/C =9.45416 + 0.77505*h_log_pbo + 1.24990*KierFlex − 0.03382*Q_VSA_HYD − 0.01507*SlogP_VSA7 − 0.03849*vsa_pol (1)

The five descriptors were determined automatically by the AutoQSAR application among the 260 calculated and displayed in the QSAR model returned. They correlate with logP (octanol/water partition coefficient) (SlogP_VSA7), the strength of π-electron bonds (h_log_pbo), the total hydrophobic van der Waals surface area (Q_VSA_HYD), the polar van der Waals surface area (vsa_pol) and the molecular flexibility (KierFlex), thus identifying these 2D molecular parameters as important for the biological activity of the DBZDs. For each descriptor, the single code detailed description and the relative importance are reported in Table 1.

The correlations among the chosen descriptors were checked and are reported in Table 2. Lack of correlation values higher than 0.7 confirms no mutual correlation among the descriptors used. In addition, calculated 2D descriptors that were not part of the final QSAR model but are considered by default important to describe the drug-likeness of molecules are reported in Table S4. No evaluation of PK profile or ADMET properties was conducted.

**Figure 1:**
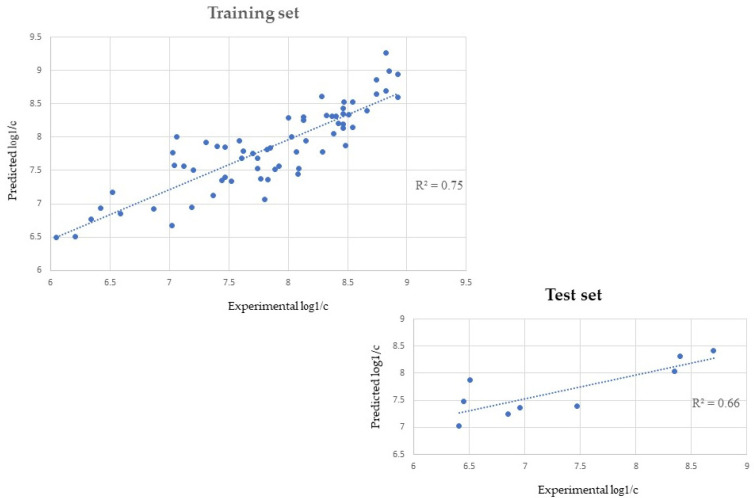
Correlation values (r^2^) for the training and the test set were obtained using the 5-descriptors QSAR model generated by AutoQSAR. Note: In the two graphs, the values of log1/c experimentally derived (x axis) are plotted against the values predicted by the QSAR model (y axis). The r^2^ defines the goodness of fit of the QSAR model. A QSAR model is considered acceptable when it has an r^2^ value > 0.6 for the training set, and an r^2^ > 0.5 for the test set. This model has, respectively, an r^2^ of 0.75 and 0.66 for the training and test set.

The predicted log1/c values for training and test set are listed in the supplementary information (Table S3), while the correlation between experimental and predicted data are visualised in Figure 1.

The chosen QSAR model was then used to predict log1/c values for the 101 DBZDs identified by NPS*finder*^®^ (Table S5).

**Figure 2:**
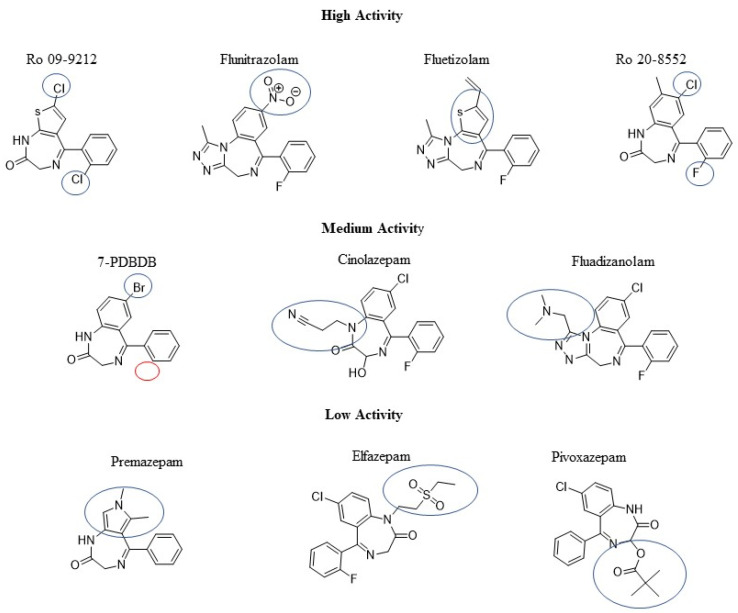
Example of 2D molecules belonging to the high, medium and low activity groups. Notes: In the figure, common chemical features across each activity bin are highlighted with a blue circle. The red circle instead indicates the lack of a substituent.

The molecules were divided into three biological activity groups according to the resulting log1/c values: low (5.80–6.99), medium (7.00–7.99) and high (>= 8.00). The number of DBZDs showing predicted log1/c values between 7.00–7.99 was 43 (42%), while the ones showing predicted log1/c > 8.00 were 42 (41%). Seventeen molecules (17%) showed predicted log1/c values lower than 7.00, indicating a low predicted biological activity. These three groups were visually analysed to possibly identify common chemical features. In the “high activity” group were observed: the recurrent presence of concomitant substitutions in position C7 and C2′, primarily with Cl, Br and NO_2_; the use of a substitute thiazole ring instead of the benzene ring in the core structure; the presence of a triazolo/imidazole ring (N1-C2) fused on the core structure (Figure 2). For the “medium activity” group observed were: the presence of one substituent only, primarily Br and F, either in position C2′ or C7; the presence of bulky substituents either in position N1 or attached to the fused imidazo/triazole ring; the lack of the pendant phenyl ring linked to the benzodiazepine core structure (Figure 2). For the “low activity” group observed were the presence of substituents in N1 and the substitution of the core structure benzene with pyrrole or imidazole rings (Figure 2). Although some chemical features are identifiable more often in only one of the three activity groups, a defined pattern/correlation could not be established.

**Figure 3:**
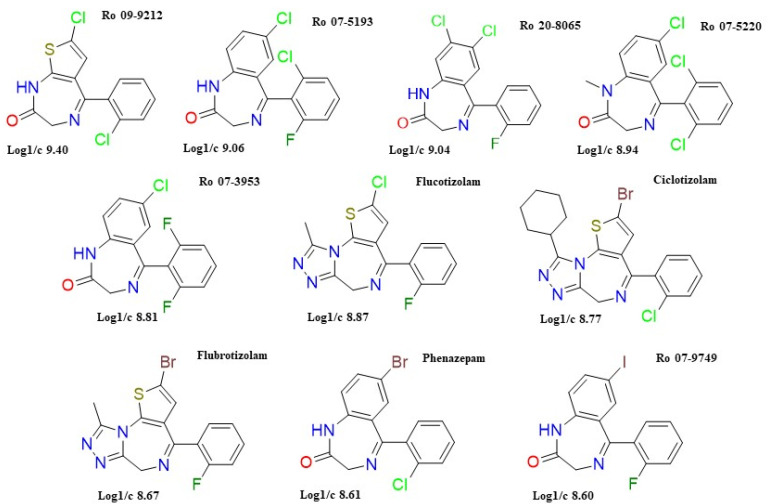
2D structures of the first ten molecules ranked according to predicted log1/c values. Colours have been used to facilitate the identification of heteroatoms (Br, Cl, I, N, O, S) in the chemical structures. The 2D structures were built with the use of ChemDraw Plus 12.0.

For the scope of this paper, only the first ten molecules (Figure 3) with the highest predicted log1/c, hence high predicted biological activity, were used for docking studies.

### 2.2. Docking

From the PDB database available structures, pdb codes 6HUO [60,61] and 6HUP [60,62] were chosen for docking studies. Both are crystallised structures of the α1β2γ3 human GABA-AR in complex with a benzodiazepine in its biologically active conformation (see methods section), respectively alprazolam and diazepam.

**Figure 4:**
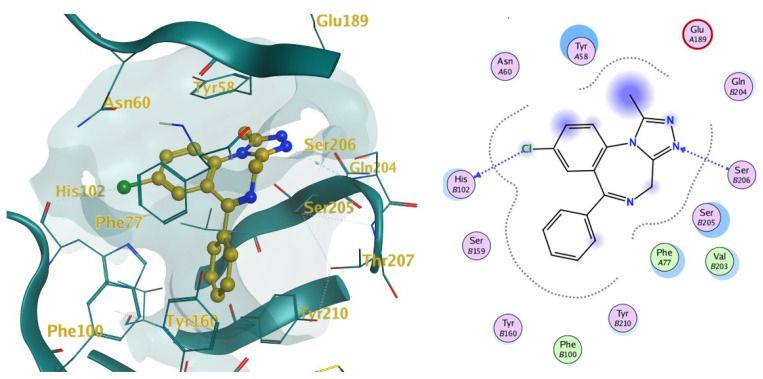
6HUO [61] binding site 3D and 2D representations. Notes: on the left, the binding pocket 3D representation. The light blue portion represents the receptor backbone whilst the golden one is the complexed ligand alprazolam. The surface of the binding pocket was added (transparent light blue) to define the size of the whole binding site. On the right, the 2D representation of the binding pocket and interactions between receptors residues and ligand is provided. This figure was generated with MOE.

These two co-crystallised structures were used as superposition targets for docking calculations and ligand interactions were explored. The 6HUO binding pocket as visualised in MOE is presented in Figure 4.

**Figure 5:**
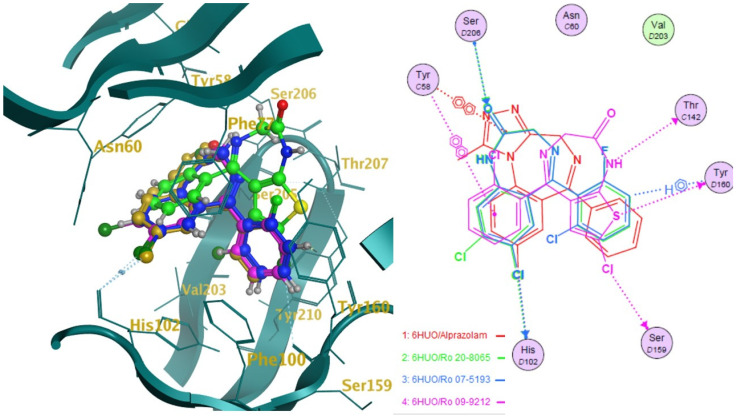
Docking studies on 6HUO [61] binding pocket. Notes: on the left side, the 3D representation of the molecules (Ro 09-9212, Ro 07-5193, Ro 20-8865) docked on the co-crystallised ligand (alprazolam in gold). On the right side is the 2D representation of the ligands’ interactions with the residues of the binding pockets. The dotted lines indicate a hydrogen bond (donor or acceptor according to the direction of the arrow at the end of it) between the ligand and the pocket residues; the dotted lines showing the H and benzene symbol indicate an arene-hydrogen interaction, while the ones showing the two benzene symbolise an arene-arene interaction.

**Figure 6:**
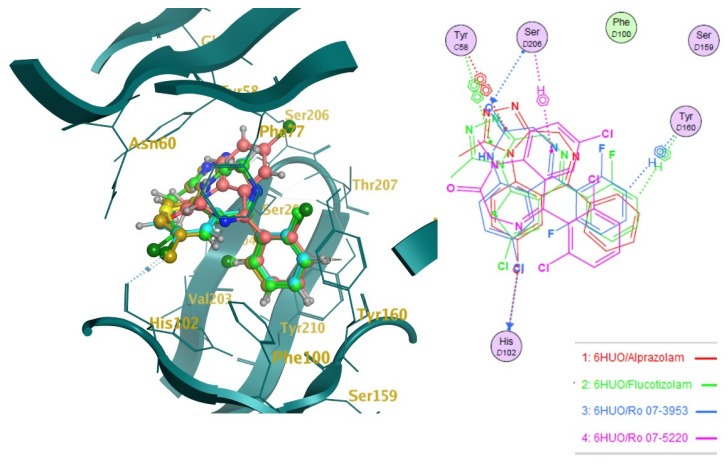
Docking studies on 6HUO [61] binding pocket. Notes: on the left side, the 3D representation of the molecules (Ro 07-3953, Ro 07-5220) docked on the co-crystallised ligand (alprazolam in gold). On the right side is the 2D representation of the ligands’ interactions with the residues of the binding pockets. The dotted lines indicate a hydrogen bond (donor or acceptor according to the direction of the arrow at the end of it) between the ligand and the pocket residues; the dotted lines showing the H and benzene symbol indicate an arene-hydrogen interaction, while the ones showing the two benzene symbolise an arene-arene interaction.

**Figure 7:**
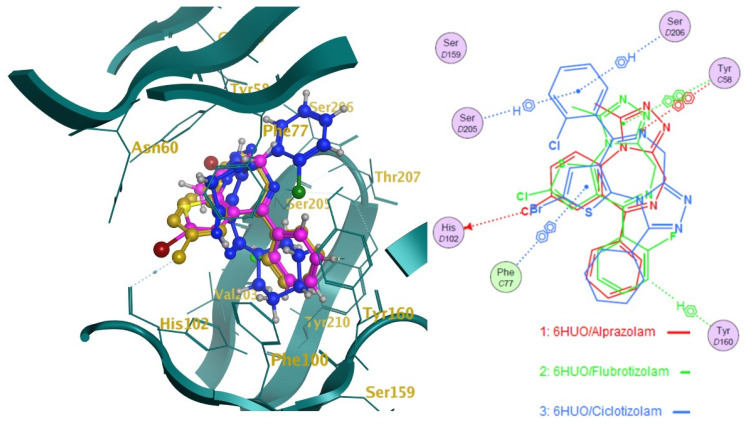
Docking studies on 6HUO [61] binding pocket. Notes: on the left side, the 3D representation of the molecules (Flubrotizolam, Ciclotizolam) docked on the co-crystallised ligand (alprazolam in gold). On the right side is the 2D representation of the ligands’ interactions with the residues of the binding pockets. The dotted lines indicate a hydrogen bond (donor or acceptor according to the direction of the arrow at the end of it) between the ligand and the pocket residues; the dotted lines showing the H and benzene symbol indicate an arene-hydrogen interaction, while the ones showing the two benzene symbolizes an arene-arene interaction.

**Figure 8:**
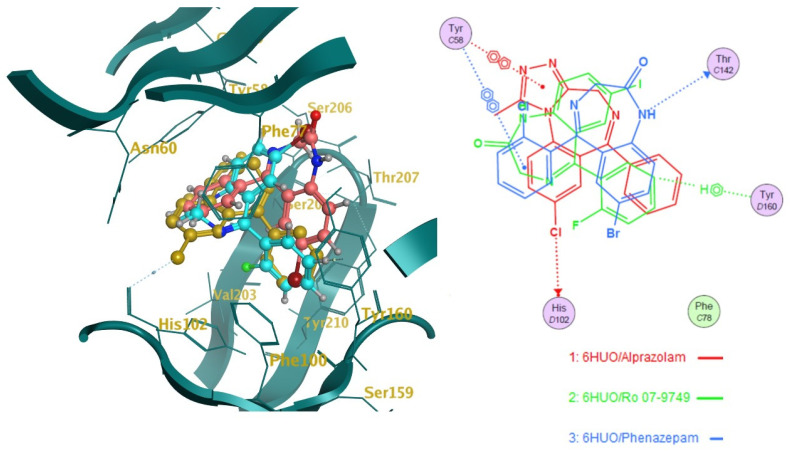
Docking studies on 6HUO [61] binding pocket. Notes: on the left side, the 3D representation of the molecules (Phenazepam, Ro 07-9749) docked on the co-crystallised ligand (alprazolam in gold). On the right side is the 2D representation of the ligands’ interactions with the residues of the binding pockets. The dotted lines indicate a hydrogen bond (donor or acceptor according to the direction of the arrow at the end of it) between the ligand and the pocket residues; the dotted lines showing the H and benzene symbol indicate an arene-hydrogen interaction, while the ones showing the two benzene symbolise an arene-arene interaction.

The ten molecules (Figure 3) that showed the highest predicted log1/c values were docked into 6HUO [61] and 6HUP [62]. London dG and GBVI/WSA dG [63] were used as the scoring methods for the docking process. For each molecule, several conformations with different S values (Kcal/mol) were returned. The ones showing the best S value (i.e., the lower the value, the more potent the binding) were identified. The same process was carried out for alprazolam and diazepam (co-crystallised ligands) and their S values were used as a reference of good binding for 6HUO and 6HUP. The S values obtained are reported in Table 3. The 3D docking pose for each of the DBZD is reported for 6HUO, as well as the 2D ligands interactions with the binding pocket residues (Figure 5, Figure 6, Figure 7 and Figure 8). As per Figure 5, Figure 6, Figure 7 and Figure 8, the most common interactions identified between the DBZD and the binding pocket are H-donor with HIS 102 (D chain, α subunit); H-pi with TYR 160 (D chain, α subunit); pi-pi with TYR 58 (C chain, γ subunit); H-acceptor with SER 206 (D chain, α subunit). The same interactions were observed with 6HUP.

### 2.3. Pharmacophore

**Figure 9:**
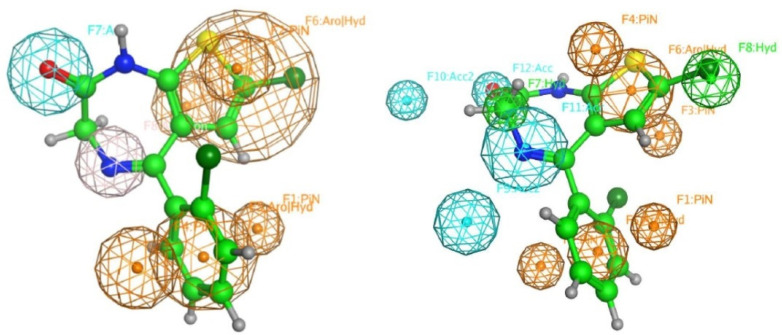
Pharmacophore queries generated for 6HUO and 6HUP. Notes: On the left side, the pharmacophore query obtained from the flexible alignment of the ten DBZDs docked in the 6HUO binding site is presented. Conversely, the one obtained from the flexible alignment of the ten DBZDs in the 6HUP binding site is provided on the right side. In both images, at the centre of the pharmacophore, and represented in green, is the molecule that showed the highest predicted value of log1/C, Ro 09-9212. Different colours were used to identify heteroatoms in the structure, specifically yellow for sulphur (S), red for oxygen (O) and blue for nitrogen (N).

The conformations with the best S values (more negative values) were further aligned to produce a pharmacophore query. The two queries for 6HUO and 6HUP [61,62] are presented in Figure 9. It should be noted that the two pharmacophore queries differed slightly i.e., the presence of an additional hydrogen feature in position C7, and an acceptor feature instead of a Donor one in C4. However, they both highlight the importance of two big aromatic functions, one in correspondence with the benzodiazepine ring and one in correspondence with the pendant phenyl ring; one hydrogen acceptor function in position C2; and one hydrogen acceptor or donor function in position C4.

## 3. Discussion

To our knowledge, this study is the first to apply computational models to a large range of DBZDs that have been identified online, with the help of an ad hoc web crawler, i.e., NPS*finder*^®^. The best 2D QSAR model obtained with MOE [63], was used here to predict the possible biological activity of previously unreported DBZDs and validated as able to predict the biological activity of new DBDZs as soon as they emerge online or on the real-world markets.

Despite being an educated guess, the data obtained by the validation of the QSAR method showed how these predicted activities can be considered reliable and useful for an initial assessment of an unknown molecule.

In fact, according to the values of r^2^ (0.75) and xr^2^ (0.69) obtained for the training set, the model generated shows very good results for the goodness-of-fit and robustness (internal validation). The goodness-of-fit, or internal predictivity (r^2^ = 0.75) indicates how well the model predicts biological activity for molecules used to build the model itself but cannot predict the efficacy of the model for compounds that were not used to train the model. Indeed, it cannot be considered as a predictivity measure for the obtained mathematical algorithm. Hence, before using the model to predict and interpret the biological activities of the DBDZs identified by NPS*finder*^®^, external validation was performed with the use of the test set. The external validation returned good values both for r^2^ (0.66) and RMSE (0.36). The r^2^ value indicates that the QSAR model was able to predict 66% of the test set activity values, hence would be able to predict the same percentage of a dataset of new DBZDs. The RSME value, instead, indicates the confidence of the model. While for the r^2^, the closer the value is to the unit the better the model works, for other external validation measures, as the error based ones (RMSE), there is no defined threshold [64]. Hence, there is no maximum value for RMSE and the lower the value the better the confidence [65,66,67].

These statistical values were obtained using the “ignore outliers” function of AutoQSAR that automatically identified an entry from the data set as a true outlier when the predicted log/c value was 2.5 units higher than the experimental one. True outliers are compounds that show unexpected biological activity or are unable to fit in a QSAR model [68]; eliminating true outliers from a training set is good practice to increase the quality of the model and avoid unnecessary bias [69]. Despite being identified with confidence by the software application, to try and explain why a compound was flagged as a true outlier, a further 3D analysis is necessary.

It should be noted that the good predictivity of this model, confirmed through internal and external validation, is efficient only on molecule classifiable as BDZs and showing the BDZ core structure, and cannot be used to predict biological activity on the GABA-Ars for other chemical classes (e.g., Z-drugs) [70]. The applicability domain that can be considered implicit here, due to the fact that only one chemical class was used to create the QSAR model, has been set so far according to structure similarity (T_c_) and include all the molecules that showed a 0.5 average T_c_ value when compared to the whole dataset used to train and validate the QSAR model.

Moreover, this QSAR model confirmed the correlation between some of the BZDs’ physiochemical characteristics already presented by previous studies [46,71,72] and biological activity. The partition coefficient (logP, i.e., membrane permeability), the hydrophobic surface, and the polar surface have been identified here as the most important parameters able to define the biological activity of an index, unknown, DBZD, together with its molecular flexibility. Although it is difficult to prove the exact correlation of each descriptor with the predicted activity, one still could speculate on their possible contribution.

In particular, Q_VSA_HYD, the total hydrophobic van der Waals (vdW) surface area appears to be the most important descriptor in the QSAR model generated (Table 1). This descriptor, together with SlogP_VSA7 (van der Waals surface area contribution to logP) and vsa_pol (vdW polar surface areas), identifies very important electrostatic molecular surface properties that are correlated with membrane solubility and have been proven to influence the membrane permeability of a molecule [73]. Moreover, hydrophobic/polar regions of a molecule could influence its position and binding in the receptor pocket according to the electrostatic distribution of the latter. KierFlex, the second most important descriptor (Table 1), indicates how the flexibility of the molecule will positively impact biological activity [74]. This may suggest the presence of a very narrow or small binding pocket in the GABA-AR that favours the binding of more flexible molecules. The sum of hydrogen bond donor strengths of carbon atoms, i.e., h_log_pbo, appears to be the third most important descriptor in the QSAR model generated (Table 1). The importance of this descriptor could be explained if one takes into consideration that the majority of the interactions documented with the docking studies (Figure 5, Figure 6, Figure 7 and Figure 8) are represented by hydrogen donor/acceptor bonds [75]. Hence the capability of a molecule to generate this type of bond could strongly correlate with its level of activity.

The other very interesting outcome of the QSAR analysis described here is the predicted values of the log1/c. As reported above, molecules with predicted values higher than 8.0 could be considered as possessing high levels of GABA-AR affinity. In fact, molecules such as lorazepam and clonazepam, which are classified as high potency prescribing BZDs, are reported in the training set with experimentally derived log1/c values respectively of 8.46 and 8.74 (Table S3).

Our prediction algorithm showed values of log1/c > 8.00 (high potency) for a big percentage (41%) of the DBZDs (*n* = 101) identified by the web crawler. Among these appear molecules such as etizolam (which is being prescribed in some countries, but still classified as DBZD in others), and flualprazolam, two of the most popular DBZDs worldwide [5,28]. These molecules reported as being responsible for multiple fatalities and near misses [76] showed predicted log 1/c values between 8.10 and 8.40. In comparison, the top ten predicted log1/c values identified (Table 3) showed putative biological activity levels up to ten times higher (log1/c = 9.40). Among the ten predicted most active DBZDs, the popular phenazepam and flucotizolam [20,21,77,78] were identified, together with less known DBZDs, of which the most potent seems to be Ro 09-9212.

Although these results need to be interpreted as only predictive values, hence not experimentally determined, they may still present a reason for concern. First, the web crawler identified 101 novel BZDs, and one could infer that further DBZDs will possibly be created in the future. Second, the QSAR results tentatively suggested that about half of these DBDZs may represent potent/very potent GABA-AR agonists. Indeed, these biological activity values are applicable to the α1 isoform of the GABA-AR, an isoform identified as being responsible for the addiction potential of BZDs [9]. Hence, one would conclude that a significant proportion of DBZDs may be both potent and associated with a strong addiction potential. Indeed, the recent scheduling of etizolam and flualprazolam [76] but also of clonazolam, diclazepam and flubromazolam [27,79] could create “vacancies” in the NPS market that could be easily filled with the range of DBZDs described here.

To investigate further the currently identified DBZDs’ predicted potency, the results from the docking studies were examined. It is important to clarify here that QSAR and docking studies are not necessarily linearly correlated, hence a high biological activity does not necessarily correspond to a high binding affinity and vice versa [80]. Furthermore, the prediction of the binding affinity for a particular substance *per se* does not give much information about the molecule/receptor interaction (e.g., agonist; partial agonist; antagonist) activity. However, docking results can be used to support QSAR analysis, hence, the predicted binding affinity values of the ten DBZDs (Table 3) were used to further analyse the predicted biological activity values. The docking (S, Kcal/mol) values obtained for alprazolam (S = −7.0) and diazepam (S = −6.6), were used as reference of a satisfactory binding affinity. As explained in the method section, alprazolam and diazepam were the active ligands co-crystallised in the receptors’ structures (6HUO and 6HUP) obtained from PDB, hence we can assume their S values to be representative of a good receptor binding. From Table 3, it is noticeable how the S values obtained from the best conformations for each of the ten most active DBZD, and for both the 6HUO and 6HUP receptors, are in line with alprazolam and diazepam. These results suggested satisfactory binding affinity levels for the α1β2γ3 human GABA-AR. Even though the docking was performed using the active conformation of two agonist BDZs (alprazolam and diazepam) as reference points, no specific information on the actual agonist/antagonist role of these DBZDs can be extrapolated.

Docking results can be very interesting in assessing the way the molecule interacts with the residues of the binding pocket (Figure 5, Figure 6, Figure 7 and Figure 8). From the 2D ligand interactions report, we can identify which residues are recurrently involved in the binding. It should be noted how the majority of the interactions happen with the α subunit of the GABA-AR binding pocket. His102 (α subunit) is confirmed again as one of the most important residues for the DBZDs binding, forming hydrogen bonds with halogenated substituents in position C_2′._ The aromatic interaction (pi-pi) between the phenyl moiety of the Tyr 58 (γ subunit) and the triazolo moiety of the DBZDs is another recurrent interaction as well as the hydrogen bonding with the Ser 206 of the α subunit. Although the receptor residues involved in the binding tend to be recurrent across the different DBDZs, the moiety of the molecule to which they bind changes. The presence of a side-chain substituent or an additional/diverse fused ring on the main core structure (e.g., triazolo, thiophene) seems to influence the orientation and positioning inside the pocket resulting in a change of the interaction pattern (Figure 5, Figure 6, Figure 7 and Figure 8). This is observable when comparing, for example, alprazolam with Ro 09-9212 (Figure 5). The presence of a thiophene ring seems to shift the molecule in the binding pocket, and the repositioning seems to be driven by the hydrogen bond between the S atom of Ro09-9212 and the Tyr 160. This results in an aromatic interaction between the pendant phenyl ring and Tyr 58, a hydrogen bond between the halogen substituent and the Ser 159, instead of the His102, and a further hydrogen bond between N_1_ and Thr 142. This change in residue interaction can be observed as well when compounds are docked in 6HUP. Further docking studies of the whole database will be carried out to understand if an interaction pattern can be identified between substituents and residues.

Finally, the best conformations resulting from the docking studies were used to retrieve a pharmacophore map of the features common to the ten DBZDs (Table 3). The resulting pharmacophore (Figure 9) confirmed the QSAR results and the importance of logP and total hydrophobic van der Waals surface area (aromatic functions in Figure 9) and the polar van der Waals surface area (hydrogen bond acceptor and donor functions in Figure 9) in defining the biological activity of a DBDZ [46,71]. The identified pharmacophore highlighted the recurring presence of both two big aromatic groups and two hydrophobic acceptor areas in those molecules showing good receptor binding affinity levels [8,81,82] and high biological activity.

### Limitations

The major limitation of this study is represented by the size of the dataset used (training and test sets) for computational studies. To obtain a robust QSAR model, a data set of roughly 100 entries would be desirable [83,84]. In this case, the small size of the dataset could have affected the QSAR predictive power. However, to the best of our knowledge, no further extensive in silico/preclinical or indeed clinical data are available. Other limitations include the use of 2D descriptors only, as well as the use of only a single software for the docking studies. When the QSAR studies started, knowledge of 3D superposition and alignment was still to be acquired hence the decision was taken to rely on 2D descriptors only. However, as including 3D descriptors could add descriptive/predictive power further study will include a 3D approach. The validity of the study is, however, supported by the consensus analysis (consistency of the findings across the three methods) of QSAR, Docking and Pharmacophore modelling that has been used for this paper [46,50].

Other limitations are linked to the fact that the NPS*finder*^®^ crawling activity and the further manual analysis has been conducted, so far, only on the surface web. Further studies from our group will focus on both the deep web and darknet [85] and will include other languages (e.g., Chinese, Japanese and Arabic). Moreover, the present NPS*finder*^®^ findings related mostly to the psychonauts’ and vendor websites, which may not represent the entirety of those NPS debated/discussed/mentioned online. Finally, the fact that a range of DBZDs are being discussed online does not necessarily mean that they are immediately accessible for purchase to interested customers.

## 4. Methods

### 4.1. Identification of Molecules

NPS*finder*^®^ automatically scanned the websites included in Table S1 between November 2017 and February 2021 and extracted the information retrieved on NPS. The predominant language used was English, but other languages were also considered: Spanish, German, Russian, Italian, Dutch, French, Swedish and Turkish. The data extracted were stored in an online, restricted access/password-controlled database and unique NPS identified and assigned to their NPS class according to their chemical structure (e.g., structure comparison with known BDZ and presence of diazepine ring) or scientific profile when available in the literature [86]. No similarity index was calculated. NPS*finder*^®^ entries were compared with the EMCDDA’s European Database on New Drugs [33] and the UNODC Early Warning Advisory on the NPS database [34]. JMC, a registered user with authorised access to these databases, prepared the listing for the comparison (Table S2). The comparison was conducted using the International Chemical Identifier Key (InChIKey) [87,88].

### 4.2. Computational Models

The computational analysis was carried out with MOE 2019.01, an integrated Computer-Aided Molecular Design Platform developed in Canada by the Chemical Computing Group ULC [63].

#### 4.2.1. QSAR

Quantitative structure-activity relationship (QSAR) models, that correlate molecular structures to biological activity, were built using physiochemical, topological, electronic and steric properties (i.e., molecular descriptors) of the molecules in question [89]. To formulate these QSAR models, a dataset of molecules whose biological activity was known and experimentally derived was built up. All the molecules, uploaded as SMILES into the MOE database, were converted to MOE molecules and underwent an energy minimisation and partial charges calculation with the molecular mechanic’s forcefields MMFF94s, using the general energy minimisation function in MOE [90]. To create the dataset, biological activity values were obtained from the literature [57]. The information used as biological activity was the logarithm of the reciprocal of concentration (log 1/c), with c being the molar inhibitory concentration (IC50) required to displace 50% of [3H]-diazepam from the rat cerebral cortex. BZDs with provisional log1/c values or atypical atoms or substituents [57] were not taken into consideration. Similarity coefficients (Tanimoto coefficient, T_c_ [91]) were calculated between all the molecules of the dataset, and average coefficients were used as a measure of similarity with the whole dataset. The average T_c_ cut off was set to 0.3, hence molecules showing values <0.3 were removed from the dataset. The latter was subsequently divided into training and test sets based on T_c_ and activity values (log1/c). On average, 80% of the dataset is included in the training set and 20% in the test set, but this ratio can be subject to modification according to the dataset size [92]. The training set was used to build the algorithm whereas the test set was used to validate it (external validation).

QSAR models were built automatically, using the partial least squares method of the AutoQSAR application in MOE. Three parameters were calculated: the correlation coefficient (r^2^) (goodness to fit) and the leave-one-out cross-validation (LOO) correlation (xr^2^) (robustness) for the training set (internal validation); and r^2^ for the test set (external validation). The r^2^ defines the goodness of fit of the QSAR model, while xr^2^ defines the goodness of prediction [58]. A QSAR model is considered acceptable when it has an r^2^ value > 0.6 and xr^2^ > 0.5 for the training set [93] and a r^2^ > 0.5 for the test set [65]. A stability test is also necessary to evaluate the significance of the model, hence the root mean square errors (RMSEs) for the training and test sets were generated [93].

The 2D descriptors (*n* = 206) were calculated and further selected according to their correlation to log1/c. The correlation was assessed with the use of QSAR-Contingency, the MOE statistical application designed for descriptors selection. The correlation to log1/c was calculated and a number between zero (no correlation) and −1 (full negative correlation) or between zero (no correlation) and 1 (full positive correlation) was obtained. Descriptors that did not correlate well or were non-contributory to the log1/c (i.e., correlation coefficient R < 0.5) were deleted. The limited cluster of descriptors obtained was then further filtered according to mutual collinearity (i.e., correlation values between one another > 0.7 resulted in rejected descriptors) and relative importance value towards log1/c values in a stepwise regression approach with repeated QSAR model generations.

Auto QSAR automatically carried out all these steps. Of the QSAR models generated, the best one was chosen according to the resulting values of r^2^, xr^2^ (i.e., closest to 1 as possible), and the number of descriptors (i.e., the lowest) used in the mathematical algorithm. QSAR rules suggest the use of roughly one descriptor for every ten molecules of the training set [92,94]. The identified final QSAR model was then validated using molecules of the test set and, once validated, used to generate predicted log1/c values for the DBZDs identified online.

#### 4.2.2. Docking

Molecular docking (MD) is also a mathematical algorithm that evaluates the binding affinity between a ligand (DBDZ) and the receptor (GABA-AR). To perform the molecular docking studies, crystallised structures of the receptor and its ligand bound in the active conformation were used and obtained from the Protein Data Bank (PDB) [95]. Available structures were refined according to source organism (the organism from which the receptor was obtained, i.e., homo sapiens,); taxonomy (i.e., eukaryote); experimental method (the technique used to generate the 3D structure, X-ray diffraction, electron microscopy, etc.); and refinement resolution (the resolution obtained in the 3D structure measured in Angstrom (Å)). In particular the CryoEM structure of human full-length alpha1beta3gamma2L GABA(A)R in complex with alprazolam, 6HUO [61], and diazepam, 6HUP [62], were chosen [60]. These structures can be identified as well in the EMDataResource database respectively as EMD-0283 [96] and EMD-0282 [97]. The chosen 3D structures were prepared with the Quick Prep MOE function. The active site identified by the co-crystallised ligands was used to dock the BDZs included in the training set to evaluate the MOE placement and scoring methods. This process involved positioning various conformations (energetically reasonable 3D atomic configurations) of the molecules with respect to the binding pocket and determining the optimal interaction geometry (the conformation of the ligand that best interacts with the ligand pocket) and its associated energy (each conformation has an energy status associated resulting from the orientation of the molecule chemical bonds). For each of these optimal interactions, a final score (S (Kcal/mol)) was returned. The lower the S value was, the stronger the predicted binding affinity of the index DBZD [63]. Once the placement and scoring methods were chosen, they were used to dock the DBZDs identified by NPS*finder*^®^.

#### 4.2.3. Pharmacophore Mapping

A pharmacophore mapping exercise was conducted on the 3D conformations obtained from the molecular docking studies. The purpose of this exercise was to define pharmacophore features common to those unknown DBDZs predicted to display the highest biological activity values. For each of these, the conformation obtained with the docking studies showing the lowest (more negative) S values was used for flexible alignment in MOE. The flexible alignment application was run on these known conformations (i.e., 3D coordinates), a set of alignments computed, and a final score returned for each of the flexible alignments generated. The final score (S) quantifies the quality of the alignment in terms of both internal strain (U score) and overlap of molecular features (F value). Lower values are intended to indicate better alignments. For the purpose of this study, the default setting was used, and the force field was changed to MMFF94. From the list of returned alignments, the one showing the lowest S value was analysed and used to generate a pharmacophore query. The pharmacophore editor application was used in the consensus mode. The unified annotation scheme was used to automatically assign pharmacophore annotation points (e.g., H-bond donor, H-bond acceptor, etc.) to the 3D conformations of the DBZDs. For each annotation point (i.e., pharmacophore feature) a percentage is returned to indicate how common that particular feature is to the set of molecules analysed. Only the features common to 70% or more of all the DBZDs were selected and displayed in the final pharmacophore description.

## 5. Conclusions

As reported in the latest UNODC reports [19,23,24], whilst DBZDs represent only a small fraction (2%) of the total number of identified NPS, they are molecules of strong interest for intravenous drug misusers, being associated with fatalities worldwide [16,24]. Indeed, they are increasingly being reported in polydrug consumption scenarios, usually with other central nervous system depressants (e.g., opiates and opioids) or stimulants. The concomitant use of more than one substance, especially of strong depressants, usually leads to a synergistic enhancement of the adverse effects of both substances, potentially leading to extremely severe side-effects including respiratory depression and death.

It is, therefore, relevant to assess as much as possible the extent of the DBZDs phenomenon. Current findings confirmed previous studies, highlighting the importance of web-based analysis [29,30,31,32] to retrieve as much information as possible relating to the current drug scenarios. Indeed, 101 DBZDs, a figure three times higher than the one reported by both the UNODC and EMCDAA was identified by this study. Currently, the UNODC [34] and the EMCDDA report a total of 30 officially identified DBZDs [5], 70% of which were identified since 2015.

As, for most DBZDs being identified, the range of relevant pharmacological and toxicity data is lacking [38], it is felt that the current findings may help in better discriminating between the different DBZDs. This is particularly true for benzodiazepine NPS because, despite their structural and chemical similarity, large differences exist between their pharmacokinetic parameters and so they are not easily comparable [12]. Indeed, it is suggested that QSAR and docking studies could be of great advantage in obtaining quick and reliable predictions on biological activity, potency and even provide initial toxicity information. In this way, it will be easier to better understand the possible harms associated with index novel DBZDs, and this may constitute a starting point for further investigations (e.g., *de novo* chemical synthesis; in vitro studies; preclinical studies).

## Figures and Tables

**Table 1:** Specification of the five descriptors included in the final QSAR model.

Code	Description	RI
h_log_pbo	Sum of log (1 + pi bond order) for all bonds	0.65
KierFlex	Kier molecular flexibility index: (KierA1) (KierA2)/n	0.68
Q_VSA_HYD	Total hydrophobic van der Waals surface area	1.00
SlogP_VSA7	approximate accessible van der Waals surface area contribution to logP(o/w)	0.25
vsa_pol	Approximation to the sum of VDW surface areas (Å2) of polar atoms	0.29

**Table 2:** Mutual correlation values for the 5 descriptors chosen for the QSAR equation.

	KierFlex	h_log_pbo	Q_VSA_HYD	vsa_pol	SlogP_VSA7
KierFlex	1.00				
h_log_pbo	0.13	1.00			
Q_VSA_HYD	0.65	0.30	1.00		
vsa_pol	0.07	0.11	−0.12	1.00	
SlogP_VSA7	−0.25	0.53	−0.09	0.07	1.00

**Table 3:** Binding values S (kcal/mol) were generated for the ten molecules that showed the highest predicted values of log1/c (nM), docked within the two receptors 6HUP and 6HUO. Notes: The molecules are listed in decreasing order of their predicted log1/c (biological activity); alprazolam and diazepam are prescription medications and here included only as a reference because they are the respective bound ligands of 6HUO and 6HUP.

Mol.	Pred. log1/c	S 6HUP (kcal/mol)	S 6HUO (Kcal/mol)
Ro 09-9212	9.40	–6.7	–6.4
Ro 07-5193	9.06	–6.7	–6.7
Ro 20-8065	9.04	–6.8	–6.6
Ro 07-5220	8.95	–6.7	–6.6
Ro 07-3953	8.81	–6.4	–6.5
Flucotizolam	8.77	–6.6	–6.8
Ciclotizolam	8.77	–7.7	–7.4
Desmethylflunitrazepam (Ro 05-4435)	8.70	–7.0	–6.6
Flubrotizolam	8.67	–6.5	–6.5
Phenazepam	8.61	–6.7	–6.6
Alprazolam	7.70	–7.0	–7.0
Diazepam	7.50	–6.6	–6.5

## Data Availability

Data is contained within the article and supplementary material.

## Supplementary Materials

The following are available online at https://www.mdpi.com/article/10.3390/ph14080720/s1, Table S1: List of websites monitored by the NPSfinder^®^ web crawler, November 2017- November 2020, surface web only; Table S2: List of the designer benzodiazepines officially reported by the UNODC and EMCDDA (February 2021); Table S3: Composition of the training and test set used to build the QSAR model; Table S4: Drug-like descriptors for the 101 DBZDs identified by the NPSfinder; Table S5: Predicted value of biological activity (log1/c) for the 101 DBZDs identified by NPSfinder^®^.

## References

[1] EMCDDA Benzodiazepines Drug Profile. http://www.emcdda.europa.eu/publications/drug-profiles/benzodiazepines_en.

[2] UNODC (2013). The International Drug Control Conventions.

[3] UNODC (2020). Schedules of the Convention on Psychotropic Substances of 1971, as at 3 November 2020.

[4] EMCDDA (2018). Perspectives on Drugs: The Misuse of Benzodiazepines among High-Risk Opioid Users in Europe.

[5] EMCDDA (2020). New Psychoactive Substances: Global Markets, Glocal Threats and the COVID-19 Pandemic—An Update from the EU Early Warning System.

[6] Griffin C.E., Kaye A.M., Rivera Bueno F., Kaye A.D. (2013). Benzodiazepine pharmacology and central nervous system-mediated effects. Ochsner J..

[7] Kelly M.D., Smith A., Banks G., Wingrove P., Whiting P.W., Atack J., Seabrook G.R., Maubach K.A. (2002). Role of the histidine residue at position 105 in the human α5 containing GABA A receptor on the affinity and efficacy of benzodiazepine site ligands. Br. J. Pharmacol..

[8] Davies M., Bateson A.N., Dunn S.M.J. (2002). Structural Requirements for Ligand Interactions at the Benzodiazepine Recognition Site of the GABAA Receptor. J. Neurochem..

[9] Tan K.R., Rudolph U., Lüscher C. (2011). Hooked on benzodiazepines: GABAA receptor subtypes and addiction. Trends Neurosci..

[10] Rudolph U., Crestani F., Benke D., Brünig I., Benson J.A., Fritschy J.M., Martin J.R., Bluethmann H., Möhler H. (1999). Benzodiazepine actions mediated by specific γ-aminobutyric acid(A) receptor subtypes. Nature.

[11] Tan K.R., Brown M., Labouébe G., Yvon C., Creton C., Fritschy J.M., Rudolph U., Lüscher C. (2010). Neural bases for addictive properties of benzodiazepines. Nature.

[12] Manchester K.R., Lomas E.C., Waters L., Dempsey F.C., Maskell P.D. (2018). The emergence of new psychoactive substance (NPS) benzodiazepines: A review. Drug Test. Anal..

[13] LSS/RAB/DPA/UNODC (2016). New Psychoactive Substances: Overview of Trends, Challenges and Legal Approaches.

[14] Drug Enforcement Administration (2019). Benzodiazepines.

[15] Orsolini L., Corkery J.M., Chiappini S., Guirguis A., Vento A., De Berardis D., Papanti D., Schifano F. (2020). “New/Designer Benzodiazepines”: An analysis of the literature and psychonauts’ trip reports. Curr. Neuropharmacol..

[16] EMCDDA (2021). New Benzodiazepines in Europe—A Review.

[17] EMCDDA (2021). European Drug Report 2021: Trends and Developments.

[18] UNODC (2020). Drug Supply World Drug Report 2020.

[19] UNODC (2021). Booklet 2—Global Overview of Drug Demand and Drug Supply.

[20] ACMD (2020). Novel Benzodiazepines A Review of the Evidence of Use and Harms of Novel Benzodiazepines.

[21] UNODC (2017). Non-Medical Use of Benzodiazepines: A Growing Threat to Public Health ?.

[22] Carpenter J.E., Murray B.P., Dunkley C., Kazzi Z.N., Gittinger M.H. (2019). Designer benzodiazepines: A report of exposures recorded in the National Poison Data System, 2014–2017. Clin. Toxicol..

[23] UNODC (2020). Current NPS Threaths Volume II.

[24] UNODC (2020). Current NPS Threats Volume III.

[25] Greenblatt H.K., Greenblatt D.J. (2019). Designer Benzodiazepines: A Review of Published Data and Public Health Significance. Clin. Pharmacol. Drug Dev..

[26] Brunetti P., Giorgetti R., Tagliabracci A., Huestis M.A., Busardò F.P. (2021). Designer Benzodiazepines: A Review of Toxicology and Public Health Risks. Pharmaceuticals.

[27] World Health Organization (2020). Summary Assessment and Recommendations of the 43rd Summary Assessment and Recommendations of the 43rd Expert Committee on Drug Dependence.

[28] Public Health England (2020). Evidence of Harm from Illicit or Fake Benzodiazepines.

[29] Schifano F., Napoletano F., Arillotta D., Zangani C., Gilgar L., Guirguis A., Corkery J.M., Vento A. (2020). The clinical challenges of synthetic cathinones. Br. J. Clin. Pharmacol..

[30] Zangani C., Schifano F., Napoletano F., Arillotta D., Gilgar L., Guirguis A., Corkery J.M., Gambini O., Vento A. (2020). The e-Psychonauts’ ‘Spiced’ World; Assessment of the Synthetic Cannabinoids’ Information Available Online. Curr. Neuropharmacol..

[31] Arillotta D., Schifano F., Napoletano F., Zangani C., Gilgar L., Guirguis A., Corkery J.M., Aguglia E., Vento A. (2020). Novel Opioids: Systematic Web Crawling within the e-Psychonauts’ Scenario. Front. Neurosci..

[32] Catalani V., Arillotta D., Corkery J.M., Guirguis A., Vento A., Schifano F. (2021). Identifying new/emerging psychoactive substances at the time of COVID-19; a web-based approach. Front. Psychiatry.

[33] EMCDDA European Database on New Drugs. https://ednd2.emcdda.europa.eu.

[34] UNODC Early Warning Advisory (EWA) on New Psychoactive Substances (NPS). https://www.unodc.org/LSS/Home/NPS.

[35] Corazza O., Assi S., Simonato P., Corkery J., Bersani F.S., Demetrovics Z., Stair J., Fergus S., Pezzolesi C., Pasinetti M. (2013). Promoting innovation and excellence to face the rapid diffusion of novel psychoactive substances in the EU: The outcomes of the ReDNet project. Hum. Psychopharmacol..

[36] Schifano F., Orsolini L., Duccio Papanti G., Corkery J.M. (2015). Novel psychoactive substances of interest for psychiatry. World Psychiatry.

[37] El Balkhi S., Chaslot M., Picard N., Dulaurent S., Delage M., Mathieu O., Saint-Marcoux F. (2017). Characterization and identification of eight designer benzodiazepine metabolites by incubation with human liver microsomes and analysis by a triple quadrupole mass spectrometer. Int. J. Legal Med..

[38] El Balkhi S., Monchaud C., Herault F., Géniaux H., Saint-Marcoux F. (2020). Designer benzodiazepines’ pharmacological effects and potencies: How to find the information. J. Psychopharmacol..

[39] Orsolini L., Francesconi G., Papanti D., Giorgetti A., Schifano F. (2015). Profiling online recreational/prescription drugs’ customers and overview of drug vending virtual marketplaces. Hum. Psychopharmacol..

[40] Orsolini L., St John-Smith P., McQueen D., Papanti D., Corkery J., Schifano F. (2017). Evolutionary Considerations on the Emerging Subculture of the E-psychonauts and the Novel Psychoactive Substances: A Comeback to the Shamanism?. Curr. Neuropharmacol..

[41] Schifano F. (2020). Coming off prescribed psychotropic medications: Insights from their use as recreational drugs. Psychother. Psychosom..

[42] Schifano F., Chiappini S., Catalani V., Napoletano F., Arillotta D., Zangani C., Guirguis A., Vento A.E., Bonaccorso S., Corkery J.M. (2020). Psychobiological, Medical, and Psychiatric Implications of New/Novel Psychoactive Substance (NPS) Use. Psychobiological Issues in Substance Use and Misuse.

[43] De Luca M.A., Castelli M.P., Loi B., Porcu A., Martorelli M., Miliano C., Kellett K., Davidson C., Stair J.L., Schifano F. (2016). Native CB1 receptor affinity, intrinsic activity and accumbens shell dopamine stimulant properties of third generation SPICE/K2 cannabinoids: BB-22, 5F-PB-22, 5F-AKB-48 and STS-135. Neuropharmacology.

[44] Valerio L.G., Choudhuri S. (2012). Chemoinformatics and chemical genomics: Potential utility of in silico methods. J. Appl. Toxicol..

[45] Artemenko A.G., Kuz’Min V.E., Muratov E.N., Polishchuk P.G., Borisyuk I.Y., Golovenko N.Y. (2009). Influence of the structure of substituted benzodiazepines on their pharmacokinetic properties. Pharm. Chem. J..

[46] Waters L., Manchester K.R., Maskell P.D., Haegeman C., Haider S. (2018). The use of a quantitative structure-activity relationship (QSAR) model to predict GABA-A receptor binding of newly emerging benzodiazepines. Sci. Justice.

[47] Durdagi S., Kapou A., Kourouli T., Andreou T., Nikas S.P., Nahmias V.R., Papahatjis D.P., Papadopoulos M.G., Mavromoustakos T. (2007). The application of 3D-QSAR studies for novel cannabinoid ligands substituted at the C1′ position of the alkyl side chain on the structural requirements for binding to cannabinoid receptors CB1 and CB2. J. Med. Chem..

[48] Durdagi S., Papadopoulos M.G., Papahatjis D.P., Mavromoustakos T. (2007). Combined 3D QSAR and molecular docking studies to reveal novel cannabinoid ligands with optimum binding activity. Bioorganic Med. Chem. Lett..

[49] Floresta G., Apirakkan O., Rescifina A., Abbate V. (2018). Discovery of high-affinity cannabinoid receptors ligands through a 3D-QSAR ushered by scaffold-hopping analysis. Molecules.

[50] Floresta G., Rescifina A., Abbate V. (2019). Structure-based approach for the prediction of mu-opioid binding affnity of unclassified designer fentanyl-like molecules. Int. J. Mol. Sci..

[51] Jia X., Ciallella H.L., Russo D.P., Zhao L., James M.H., Zhu H. (2021). Construction of a Virtual Opioid Bioprofile: A Data-Driven QSAR Modeling Study to Identify New Analgesic Opioids. ACS Sustain. Chem. Eng..

[52] Zhang Z., An L., Hu W., Xiang Y. (2007). 3D-QSAR study of hallucinogenic phenylalkylamines by using CoMFA approach. J. Comput. Aided. Mol. Des..

[53] Schulze-Alexandru M., Kovar K.-A., Vedani A. (1999). Quasi-atomistic Receptor Surrogates for the 5-HT2A Receptor: A 3D-QSAR Study on Hallucinogenic Substances. Mol. Inform..

[54] Wu N., Feng Z., He X., Kwon W., Wang J., Xie X.Q. (2019). Insight of Captagon Abuse by Chemogenomics Knowledgebase-guided Systems Pharmacology Target Mapping Analyses. Sci. Rep..

[55] Guariento S., Tonelli M., Espinoza S., Gerasimov A.S., Gainetdinov R.R., Cichero E. (2018). Rational design, chemical synthesis and biological evaluation of novel biguanides exploring species-specificity responsiveness of TAAR1 agonists. Eur. J. Med. Chem..

[56] Schifano F., Chiappini S., Miuli A., Corkery J.M., Scherbaum N., Napoletano F., Arillotta D., Zangani C., Catalani V., Vento A. (2021). New psychoactive substances (NPS) and serotonin syndrome onset: A systematic review. Exp. Neurol..

[57] Hadjipavlou-Litina D., Hansch C. (1994). Quantitative Structure‒Activity Relationships of the Benzodiazepines. A Review and Reevaluation. Chem. Rev..

[58] Golbraikh A., Tropsha A. (2000). Predictive QSAR modeling based on diversity sampling of experimental datasets for the training and test set selection. Mol. Divers..

[59] Roy K., Das R.N., Ambure P., Aher R.B. (2016). Be aware of error measures. Further studies on validation of predictive QSAR models. Chemom. Intell. Lab. Syst..

[60] Masiulis S., Desai R., Uchański T., Serna Martin I., Laverty D., Karia D., Malinauskas T., Zivanov J., Pardon E., Kotecha A. (2019). GABAA receptor signalling mechanisms revealed by structural pharmacology. Nature.

[61] RCSB PDB 6HUO: CryoEM Structure of Human Full-Length Heteromeric alpha1beta3gamma2L GABA(A)R in Complex with Alprazolam (Xanax), GABA and Megabody Mb38. https://www.rcsb.org/structure/6HUO.

[62] RCSB PDB 6HUP: CryoEM Structure of Human Full-Length alpha1beta3gamma2L GABA(A)R in Complex with Diazepam (Valium), GABA and megabody Mb38. https://www.rcsb.org/structure/6HUP.

[63] Chemical Computing Group ULC (2021). Molecular Operating Enviroment (MOE), 2019.01.

[64] Consonni V., Ballabio D., Todeschini R. (2010). Evaluation of model predictive ability by external validation techniques. J. Chemom..

[65] Golbraikh A., Tropsha A. (2002). Beware of q2!. J. Mol. Graph. Model..

[66] Alexander D.L.J., Tropsha A., Winkler D.A. (2015). Beware of R2: Simple, Unambiguous Assessment of the Prediction Accuracy of QSAR and QSPR Models. J. Chem. Inf. Model..

[67] Worachartcheewan A., Toropova A.P., Toropov A.A., Siriwong S., Prapojanasomboon J., Prachayasittikul V., Nantasenamat C. (2018). Quantitative Structure-activity Relationship Study of Betulinic Acid Derivatives Against HIV using SMILES-based Descriptors. Curr. Comput. Aided. Drug Des..

[68] Verma R.P., Hansch C. (2005). An approach toward the problem of outliers in QSAR. Bioorganic Med. Chem..

[69] Furusjö E., Svenson A., Rahmberg M., Andersson M. (2006). The importance of outlier detection and training set selection for reliable environmental QSAR predictions. Chemosphere.

[70] Gunja N. (2013). The Clinical and Forensic Toxicology of Z-drugs. J. Med. Toxicol..

[71] Thakur M., Thakur A., Sudele P. (2004). Comparative QSAR and QPAR study of benzodiazepines. Indian J. Chem..

[72] Maddalena D.J., Johnston G.A.R. (1995). Prediction of Receptor Properties and Binding Affinity of Ligands to Benzodiazepine/Gm& Receptors Using Artificial Neural Networks. J. Med. Chem.

[73] Wildman S.A., Crippen G.M. (1999). Prediction of physicochemical parameters by atomic contributions. J. Chem. Inf. Comput. Sci..

[74] Hall L.H., Kier L.B. (1991). The Molecular Connectivity Chi Indexes and Kappa Shape Indexes in Structure-Property Modeling.

[75] Wang Y., Liu H., Fan Y., Chen X., Yang Y., Zhu L., Zhao J., Chen Y., Zhang Y. (2019). In Silico Prediction of Human Intravenous Pharmacokinetic Parameters with Improved Accuracy. J. Chem. Inf. Model..

[76] UNODC EWA March 2020- Recently Scheduled Benzodiazepines Flualprazolam and Etizolam Associated with Multiple Post-mortem and DUID Cases in UNODC EWA. https://www.unodc.org/LSS/Announcement/Details/ad0c279b-b4d4-49f3-b638-cd87755d2d42.

[77] Moosmann B., Auwärter V. (2018). Designer benzodiazepines: Another class of new psychoactive substances. Handbook of Experimental Pharmacology.

[78] WHO (2015). Phenazepam Pre-Review Report Agenda Item 5.8 Expert Committee on Drug Dependence Thirty-seventh Meeting.

[79] UNODC WHO: World Health Organization Recommends 8 NPS for Scheduling. https://www.unodc.org/LSS/Announcement/Details/0d68dc5f-a17e-4edc-83f0-6705aca1e5b3.

[80] Chen Y.C. (2015). Beware of docking!. Trends Pharmacol. Sci..

[81] Sigel E., Ernst M. (2018). The Benzodiazepine Binding Sites of GABAA Receptors. Trends Pharmacol. Sci..

[82] Sigel E., Luscher B.P. (2012). A Closer Look at the High Affinity Benzodiazepine Binding Site on GABAA Receptors. Curr. Top. Med. Chem..

[83] Golbraikh A., Muratov E., Fourches D., Tropsha A. (2014). Data set modelability by QSAR. J. Chem. Inf. Model..

[84] Fourches D., Muratov E., Tropsha A. (2010). Trust, but verify: On the importance of chemical structure curation in cheminformatics and QSAR modeling research. J. Chem. Inf. Model..

[85] Orsolini L., Papanti D., Corkery J., Schifano F. (2017). An insight into the deep web; why it matters for addiction psychiatry?. Hum. Psychopharmacol..

[86] Schifano F., Napoletano F., Chiappini S., Guirguis A., Corkery J.M., Bonaccorso S., Ricciardi A., Scherbaum N., Vento A. (2019). New/emerging psychoactive substances and associated psychopathological consequences. Psychol. Med..

[87] Heller S., McNaught A., Stein S., Tchekhovskoi D., Pletnev I. (2013). InChI - The worldwide chemical structure identifier standard. J. Cheminform..

[88] Mcnaught A. (2006). The IUPAC International Chemical Identifier: InChl-A New Standard for Molecular Informatics. Chem. Int..

[89] Gad S.C., Wexler P. (2014). QSAR. Encyclopedia of Toxicology: Third Edition.

[90] Halgren T.A. (1999). MMFF VI. MMFF94s option for energy minimization studies. J. Comput. Chem..

[91] Bajusz D., Rácz A., Héberger K. (2015). Why is Tanimoto index an appropriate choice for fingerprint-based similarity calculations?. J. cheminformatics.

[92] Leelananda S.P., Lindert S. (2016). Computational methods in drug discovery. Beilstein J. Org. Chem..

[93] Beebe R.K., Pell J.R., Seasholts M.B. (1998). Chemometrics: A Practical Guide.

[94] Tropsha A. (2010). Best Practices for QSAR Model Development, Validation, and Exploitation. Mol. Inform..

[95] RCSB PDB: Homepage. https://www.rcsb.org/.

[96] EMDataResource EMDR: EMD-0283. https://www.emdataresource.org/EMD-0283.

[97] EMDataResource EMDR: EMD-0282. https://www.emdataresource.org/EMD-0282.

