# Supplementary material for: The Psychonauts’ Benzodiazepines; Quantitative Structure-Activity Relationship (QSAR) Analysis and Docking Prediction of Their Biological Activity
Source: Pharmaceuticals (Basel). 2021 Jul 26;14(8):720. doi: 10.3390/ph14080720 (PMC8398354; doi:10.3390/ph14080720)
Supplement: Supplementary file 1 [file pharmaceuticals-14-00720-s001.zip › pharmaceuticals-1312155-supplementary/Table S1.pdf]

Table S1: List of websites monitored by the NPSfinder® web crawler, November 2017- November 2020, surface web only.

N	Website name
**1**	Avalonmagicplants.com
**2**	Azarius.net
**3**	Bluelight.org
**4**	Bluemorphotours.com
**5**	Cannabis.net
**6**	Chemeurope.com
**7**	Committedpsychonaut.tumblr.com
**8**	Consolidated Index of Controlled Substances
**9**	Daath.hu/psychonauts
**10**	Dancesafe.org
**11**	Deviantart.com/psychonaut-a
**12**	Druglibrary.org
**13**	Drugs.tripsit.me
**14**	Drugs-forum.com
**15**	Drugs-plaza.com
**16**	Dutch-headshop.eu
**17**	Ecstasydata.org
**18**	Elephantos.com
**19**	Energycontrol.org
**20**	Entheogen-network.com/forums
**21**	Erowid.org
**22**	Eusynth.org
**23**	Everything2.com/title/Psychonaut
**24**	Fungifun.org
**25**	Hedweb.com
**26**	Hipforums.com/forum
**27**	Isomerdesign.com
**28**	Knehnav.home.xs4all.nl
**29**	Kratomshop.com
**30**	Legal-high-inhaltsstoffe.de
**31**	Mindstates.org
**32**	Mycotopia.net
**33**	Natmedtalk.com
**34**	Npsproject.eu
**35**	Peyote.com/peyolink.html
**36**	Psychedelic-library.org
**37**	Psychonaut.ca
**38**	Psychonaut.fr
**39**	Psychonautdocs.com
**40**	Psychonautwiki.org
**41**	Psyconauts.tripod.com
**42**	Reddit.com and drug-related subreddits (e.g. Reddit.com/r/Psychonaut/; Reddit.com/r/shroomers/)

**43**	Shayanashop.com
**44**	Sjamaan.com
**45**	Tripzine.com
**46**	Tryptamind.com
**47**	Urban75.net
**48**	Wikipedia List of designer drugs
**49**	Zamnesia.com
