# Supplementary material for: The Psychonauts’ Benzodiazepines; Quantitative Structure-Activity Relationship (QSAR) Analysis and Docking Prediction of Their Biological Activity
Source: Pharmaceuticals (Basel). 2021 Jul 26;14(8):720. doi: 10.3390/ph14080720 (PMC8398354; doi:10.3390/ph14080720)
Supplement: Supplementary file 1 [file pharmaceuticals-14-00720-s001.zip › pharmaceuticals-1312155-supplementary/Table S2.pdf]

Table S2: List of the designer benzodiazepines officially reported by the UNODC and EMCDDA (February 2021).

Designer Benzodiazepines	UNODC	EMCDDA
Alprazolam triazolobenzophenone derivative	Y	Y
2-ethyl-4-phenyl-6H-thieno[3,2-f][1,2,4]triazolo[4,3-a][1,4]diazepine	Y	N
3-hydroxyphenazepam	Y	Y
4-chlorodiazepam	Y	Y
Adinazolam	Y	Y
Bromazolam	Y	Y
Clobromazolam	Y	Y
Clonazolam / Clonitrazolam	Y	Y
Cloniprazepam	Y	Y
Deschloroetizolam	Y	Y
Desmethylflunitrazepam (Fonazepam)	Y	Y
Diclazepam (chlorodiazepam)	Y	Y
Etizolam	Y	Y
Flualprazolam	Y	Y
Flubromazepam	Y	Y
Flubromazolam	Y	Y
Fluclotizolam	Y	Y
Flunitrazolam	Y	Y
Meclonazepam	Y	Y
Methyl clonazepam	Y	Y
Metizolam	Y	Y
Nifoxipam	Y	Y
Nitrazolam	Y	Y
Norfludiazepam	Y	Y
Phenazepam	Y	Y
Pyrazolam	Y	Y
Ro-07-4065 (Difludiazepam)	Y	Y
Thionordazepam	Y	Y
Zapizolam	Y	N
Bentazepam	N	Y
Tofisopam	N	Y
