# Supplementary material for: The Psychonauts’ Benzodiazepines; Quantitative Structure-Activity Relationship (QSAR) Analysis and Docking Prediction of Their Biological Activity
Source: Pharmaceuticals (Basel). 2021 Jul 26;14(8):720. doi: 10.3390/ph14080720 (PMC8398354; doi:10.3390/ph14080720)
Supplement: Supplementary file 1 [file pharmaceuticals-14-00720-s001.zip › pharmaceuticals-1312155-supplementary/Table S3.pdf]

Table S3: Composition of the training and test set used to build the QSAR model The experimentally derived [57] and predicted values of log1/c for each molecules are presented as well..

Training set			
**Molecule**	**Smile**	**log 1/c**	**Pred log 1/c**
Brotizolam	Brc1sc2-n3c(C)nnc3CN=C(c3c(Cl)cccc3)c2c1	8.92	8.94
Meclonazepam	Clc1c(C2=NC(C)C(=O)Nc3c2cc([N+](=O)[O-])cc3)cccc1	8.92	8.60
Ro 11-1465	Clc1c(C2=NCc3n(c(C)nn3)-c3sc(Cl)cc23)cccc1	8.85	8.99
Ro 05-4435	Fc1c(C2=NCC(=O)Nc3c2cc([N+](=O)[O-])cc3)cccc1	8.82	8.69
Ro 14-1636	Ic1sc2-n3c(C)nnc3CN=C(c3c(Cl)cccc3)c2c1	8.82	9.26
Clonazepam	Clc1c(C2=NCC(=O)Nc3c2cc([N+](=O)[O-])cc3)cccc1	8.74	8.86
Delorazepam	Clc1c(C2=NCC(=O)Nc3c2cc(Cl)cc3)cccc1	8.74	8.65
Ro 05-4082	Clc1c(C2=NCC(=O)N(C)c3c2cc([N+](=O)[O-])cc3)cccc1	8.66	8.40
Ro 07-9957	Ic1cc2C(c3c(F)cccc3)=NCC(=O)N(C)c2cc1	8.54	8.15
Ro 11-7800	Clc1c(C2=NCc3n(c(CN)nn3)-c3sc(Cl)cc23)cccc1	8.54	8.53
Etizolam	Clc1c(C2=NCc3n(c(C)nn3)-c3sc(CC)cc23)cccc1	8.51	8.34
Ro 11-5073	Clc1cc2C(c3c(F)cccc3)=NC(C)c3n(c(C)nn3)-c2cc1	8.48	7.87
Ro 11-5074	Clc1c(C2=NC(SC)c3n(c(C)nn3)-c3c2cc([N+](=O)[O-])cc3)cccc1	8.47	8.53
Lorazepam	Clc1c(C2=NC(O)C(=O)Nc3c2cc(Cl)cc3)cccc1	8.46	8.20
Ro 05-3590	FC(F)(F)c1c(C2=NCC(=O)Nc3c2cc([N+](=O)[O-])cc3)cccc1	8.46	8.35
Ro 11-4878	Clc1cc2C(c3c(F)cccc3)=NC(C)C(=O)Nc2cc1	8.46	8.14
Ro 17-4582	Clc1c(C2=NCc3n(c(C)nn3)-c3sccc23)cccc1	8.46	8.43
Flunitrazepam	Fc1c(C2=NCC(=O)N(C)c3c2cc([N+](=O)[O-])cc3)cccc1	8.42	8.21
Ro 11-6679	S(C)C1N=C(c2c(F)cccc2)c2c(-n3c(C)nnc13)ccc([N+](=O)[O-])c2	8.40	8.31
Hydroxytriazolam	Clc1c(C2=NCc3n(c(CO)nn3)-c3c2cc(Cl)cc3)cccc1	8.38	8.05
U-35005	Clc1c(C2=NCc3n(c(C)nn3)-c3c2cccc3)cccc1	8.37	8.31
Midazolam	Clc1cc2C(c3c(F)cccc3)=NCc3n(c(C)nc3)-c2cc1	8.32	8.33
Ro 05-6822	Fc1c(C2=NCC(=O)N(C)c3c2cc(F)cc3)cccc1	8.29	7.78
Ro 20-7078	Clc1cc2C(c3c(F)cccc3)=NC(Cl)C(=O)Nc2cc1	8.28	8.61
Ro 11-6896	Fc1c(C2=NC(C)C(=O)N(C)c3c2cc([N+](=O)[O-])cc3)cccc1	8.15	7.94
Ro 05-6820	Fc1c(C2=NCC(=O)Nc3c2cc(F)cc3)cccc1	8.13	8.26
Ro 21-5205	Clc1cc2C(c3c(F)cccc3)=NCc3c(C(=O)OC)ncn3-c2cc1	8.13	8.30
Diazepam	Clc1cc2C(c3ccccc3)=NCC(=O)N(C)c2cc1	8.09	7.53
Ro 07-1986	ClN1C(=O)CN=C(c2c(F)cccc2)c2c1ccc(CCN)c2	8.08	7.45
Estazolam	Clc1cc2C(c3ccccc3)=NCc3n(-c2cc1)cnn3	8.07	7.78
Nordiazepam	Clc1cc2C(c3ccccc3)=NCC(=O)Nc2cc1	8.03	8.00
Nitrazepam	O=[N+]([O-])c1cc2C(c3ccccc3)=NCC(=O)Nc2cc1	8.00	8.29
Ro 22-1892	Clc1cc2C(c3ccccc3)=NCc3c(C(=O)OC(C)C)ncn3-c2cc1	7.92	7.56
Ro 05-2904	FC(F)(F)c1cc2C(c3ccccc3)=NCC(=O)Nc2cc1	7.89	7.52
Ro 16-0529	Clc1c2C(c3ccccc3)=NCc3c(C(=O)OCC)ncn3-c2ccc1	7.85	7.84
Flurazepam	Clc1cc2C(c3c(F)cccc3)=NCC(=O)N(CCN(CC)CC)c2cc1	7.83	7.36
Ro 15-8670	Clc1cc2C(c3ccccc3)=NCc3c(C(=O)OCC)ncn3-c2cc1	7.82	7.82
Temazepam	Clc1cc2C(c3ccccc3)=NC(O)C(=O)N(C)c2cc1	7.80	7.07
Ro 05-4865	Fc1cc2C(c3ccccc3)=NCC(=O)N(C)c2cc1	7.77	7.38
Oxazepam	Clc1cc2C(c3ccccc3)=NC(O)C(=O)Nc2cc1	7.74	7.53
Ro 20-3053	Fc1c(C2=NCC(=O)Nc3c2cc(C(=O)C)cc3)cccc1	7.74	7.69

Alprazolam	Clc1cc2C(c3ccccc3)=NCc3n(c(C)nn3)-c2cc1	7.70	7.75
Ro 20-5747	O=C1Nc2c(C(c3ccccc3)=NC1)cc(C=C)cc2	7.62	7.79
Ro 07-2750	Clc1cc2C(c3c(F)cccc3)=NCC(=O)N(CCO)c2cc1	7.61	7.68
Ro 21-8482	Clc1c(C2=NCc3c(C(=O)N)nc(C)n3-c3c2cc(Cl)cc3)cccc1	7.59	7.94
Ro 20-2541	Fc1c(C2=NCC(=O)N(C)c3c2cc(C#N)cc3)cccc1	7.52	7.34
Desmethyltetrazepam	Clc1cc2C(C3=CCCCC3)=NCC(=O)Nc2cc1	7.47	7.85
Tetrazepam	Clc1cc2C(C3=CCCCC3)=NCC(=O)N(C)c2cc1	7.47	7.40
Ro 20-2533	O=C1Nc2c(C(c3ccccc3)=NC1)cc(CC)cc2	7.44	7.35
Ro 05-3061	Fc1cc2C(c3ccccc3)=NCC(=O)Nc2cc1	7.40	7.86
Ro 08-9013	ClN1C(=O)CN=C(c2c(F)cccc2)c2c1ccc(CCOCC(=O)N)c2	7.37	7.12
Ro 06-7263	ClN1C(=O)C(C)N=C(c2ccccc2)c2c1ccc(Cl)c2	7.31	7.92
Ro 08-3026	Clc1c(C2=NCC(=O)Nc3c2cc(COCCN)cc3)cccc1	7.20	7.50
Ro 20-1815	Fc1c(C2=NCC(=O)N(C)c3c2cc(N)cc3)cccc1	7.19	6.95
Ro 05-4619	Clc1c(C2=NCC(=O)Nc3c2cc(N)cc3)cccc1	7.12	7.57
Ro 05-3328	Clc1cc2C(c3ccccc3)=NCC(=O)Nc2cc1	7.06	8.00
Halazepam	Clc1cc2C(c3ccccc3)=NCC(=O)N(CC(F)(F)F)c2cc1	7.04	7.58
Pinazepam	Clc1cc2C(c3ccccc3)=NCC(=O)N(CC#C)c2cc1	7.03	7.77
Ro 20-7736	Fc1c(C2=NCC(=O)N(C)c3c2cc(NO)cc3)cccc1	7.02	6.67
Adinazolam	Clc1cc2C(c3ccccc3)=NCc3n(c(CN(C)C)nn3)-c2cc1	6.87	6.92
Ro 17-2221	O=C1Nc2c(C(c3ccccc3)=NC1)cc(CCN)cc2	6.59	6.85
Ro 22-4683	Fc1c(C2=NCC(=O)N(C(C)(C)C)c3c2cc([N+](=O)[O-])cc3)cccc1	6.52	7.17
Ro 05-4528	O=C1N(C)c2c(C(c3ccccc3)=NC1)cc(C#N)cc2	6.42	6.94
Ro 12-6377	Fc1c(C2=NCC(=O)N(C)c3c2cc(NC(=O)NC)cc3)cccc1	6.34	6.77
Ro 20-1310	Clc1cc2C(c3ccccc3)=NCC(=O)N(C(C)(C)C)c2cc1	6.21	6.51
Camazepam	Clc1cc2C(c3ccccc3)=NC(OC(=O)N(C)C)C(=O)N(C)c2cc1	6.05	6.50
**Test set**			
**Molecule**	**Smile**	**log 1/C**	**PRED log 1/C**
Prazepam	Clc1cc2C(c3ccccc3)=NCC(=O)N(CC3CC3)c2cc1	6.96	7.36
Ro 05-2921	O=C1Nc2c(C(c3ccccc3)=NC1)cccc2	6.45	7.48
7-Aminonitrazepam	O=C1Nc2c(C(c3ccccc3)=NC1)cc(N)cc2	6.41	7.03
Norfludiazepam	Clc1cc2C(c3c(F)cccc3)=NCC(=O)Nc2cc1	8.70	8.41
Ro 05-4336	Fc1c(C2=NCC(=O)Nc3c2cccc3)cccc1	6.51	7.87
Ro 05-4520	Fc1c(C2=NCC(=O)N(C)c3c2cccc3)cccc1	7.47	7.39
Proflazepam	Clc1cc2C(c3c(F)cccc3)=NCC(=O)N(CC(O)CO)c2cc1	6.85	7.25
Triazolam	CC1=NN=C2N1C3=C(C=C(C=C3)Cl)C(=NC2)C4=CC=CC=C4Cl	8.40	8.31
4-hydroxymidazolam	Clc1cc2C(c3c(F)cccc3)=NCc3n(c(CO)nc3)-c2cc1	8.35	8.03
