# Supplementary material for: The Psychonauts’ Benzodiazepines; Quantitative Structure-Activity Relationship (QSAR) Analysis and Docking Prediction of Their Biological Activity
Source: Pharmaceuticals (Basel). 2021 Jul 26;14(8):720. doi: 10.3390/ph14080720 (PMC8398354; doi:10.3390/ph14080720)
Supplement: Supplementary file 1 [file pharmaceuticals-14-00720-s001.zip › pharmaceuticals-1312155-supplementary/Table S4.pdf]

Table S4: ***Drug-like parameters and predicted log1/c values for the 101 DBZDs identified by the NPSfinder. The parameters considered were : MW (molecular weight),TP vdw SA (Total polar vdw surface area), SlogP (Log Octanol/Water Partition Coefficient), lip_acc (Lipinski Acceptor Count), lip_don (Lipinski Donor Count), lip_druglike (Lipinski Druglike test), lip_violation (Lipinski Violation count).***

Molecule	MW	TP VDW SA	SlogP	lip_acc	lip_don	lip_druglike	lip_violation	Predicted log1/c
Ro 09-9212	311.2	58.2	3.8	3	1	1	0	9.4
Ro 07-5193	323.2	70.1	3.9	3	1	1	0	9.1
Ro 20-8065	323.2	70.1	3.9	3	1	1	0	9.0
Ro 07-5220	353.6	52.5	4.5	3	0	1	0	8.9
Ro 07-3953	306.7	82.0	3.4	3	1	1	0	8.8
Flucotizolam	332.8	17.6	4.0	4	0	1	0	8.8
Ciclotizolam	461.8	5.7	6.4	4	0	1	1	8.8
Flubrotizolam	377.2	17.6	4.2	4	0	1	0	8.7
Phenazepam	349.6	58.2	3.9	3	1	1	0	8.6
Ro 07-9749	380.2	70.1	3.2	3	1	1	0	8.6
Clonazolam	353.8	46.3	3.8	7	0	1	0	8.6
Ro 15-9270	352.8	40.6	3.9	6	0	1	0	8.5
Climazolam	342.2	11.4	5.1	3	0	1	1	8.5
Flunitrazolam	337.3	58.2	3.2	7	0	1	0	8.5
Ro 20-8552	302.7	70.1	3.6	3	1	1	0	8.4
Methyl Clonazepam	329.7	93.1	3.1	6	0	1	0	8.4
Reclazepam	374.2	74.2	3.6	5	0	1	0	8.4
Uldazepam	360.2	39.3	4.6	4	1	1	0	8.4
Zapizolam	330.2	36.2	3.6	5	0	1	0	8.4
Ethyl Dirazepate	377.2	75.4	3.7	5	1	1	0	8.4
Difludiazepam (RO- 07-4065)	320.7	76.3	3.4	3	0	1	0	8.4
Metizolam	328.8	30.5	4.2	4	0	1	0	8.4
Etizolam	342.9	5.7	4.5	4	0	1	0	8.3
Desmethyltriazolam	329.2	30.5	4.2	4	0	1	0	8.3
Flubromazepam	333.2	70.1	3.4	3	1	1	0	8.3
Ro 13-3780	365.2	76.3	3.5	3	0	1	0	8.3
Lopirazepam	322.2	56.5	2.5	5	2	1	0	8.2

Cloniprazepam	369.8	93.1	3.8	6	0	1	0	8.2
Nifoxipam (3-OH-Norflunitrazepam)	315.3	103.4	1.8	7	2	1	0	8.2
Phenazolam (Clobromazolam, DM-ii-90)	387.7	5.7	4.6	4	0	1	0	8.2
Diclazepam	319.2	52.5	3.8	3	0	1	0	8.2
4'-Chlorodiazepam	319.2	52.5	3.8	3	0	1	0	8.2
3-Hydroxyphenazepam	365.6	50.8	3.2	4	2	1	0	8.2
Bentazepam	296.4	58.2	3.4	3	1	1	0	8.2
Cinazepam	465.7	109.9	3.6	7	2	1	0	8.2
Flualprazolam	326.8	17.6	4.0	4	0	1	0	8.1
Metaclazepam	393.7	8.2	4.4	3	0	1	0	8.1
Fluetizolam	326.4	17.6	4.0	4	0	1	0	8.1
Pynazolam	320.3	52.0	2.5	8	0	1	0	8.1
Desmethylnitrazolam	305.3	71.1	2.8	7	0	1	0	8.1
Nitrazolam	319.3	46.3	3.1	7	0	1	0	8.1
Flubromazolam	371.2	17.6	4.1	4	0	1	0	8.0
Fletazepam	370.7	93.8	4.2	3	0	1	0	8.0
7-BPDBD	315.2	58.2	3.2	3	1	1	0	7.9
Tuclazepam	335.2	41.3	3.6	3	1	1	0	7.9
SH-053-R-CH3-2'F	387.4	54.1	4.2	5	0	1	0	7.8
Pyclazolam	309.8	11.4	3.2	5	0	1	0	7.8
RO 21-8137	354.8	49.8	3.4	5	2	1	0	7.8
Estazolam	294.7	30.5	3.5	4	0	1	0	7.8
Pyeazolam (SH-TRI-108)	299.3	11.4	2.6	5	0	1	0	7.8
Flutoprazepam	342.8	64.4	4.1	3	0	1	0	7.8
Rilmazolam	400.3	49.1	3.9	6	0	1	0	7.8
Deschloroetizolam	308.4	5.7	3.8	4	0	1	0	7.7
Zometapine	274.8	5.7	3.0	4	1	1	0	7.7
Pypazolam	354.2	11.4	3.4	5	0	1	0	7.6
Imidazenil	399.2	49.8	3.5	5	2	1	0	7.6
Menitrazepam	299.3	93.1	2.9	6	0	1	0	7.6
Bromazolam	353.2	5.7	4.0	4	0	1	0	7.6

MP-iii-022	372.4	55.5	3.4	5	1	1	0	7.6
Ro 05-4608	284.7	52.5	3.2	3	0	1	0	7.6
Cyprazepam	339.8	66.5	3.7	4	1	1	0	7.5
Triflunordazepam	304.3	67.2	3.8	3	1	1	0	7.5
Quazepam	386.8	26.6	5.0	2	0	1	1	7.5
N-Methylbromazepam	330.2	58.2	2.7	4	0	1	0	7.5
Flutemazepam	318.7	57.1	2.6	4	1	1	0	7.5
Thionordazepam	286.8	5.7	3.9	2	1	1	0	7.4
Iomazenil	411.2	63.0	2.5	6	0	1	0	7.4
QH-II-066	274.3	52.5	2.5	3	0	1	0	7.4
CP-1414S	296.3	72.8	2.7	7	2	1	0	7.4
Lofendazam	272.7	26.5	3.8	3	1	1	0	7.4
Tofisopam	382.5	10.0	4.4	6	0	1	0	7.4
Fluadinazolam	369.8	17.6	4.0	5	0	1	0	7.3
Remimazolam	439.3	47.8	4.3	6	0	1	0	7.3
Ethyl Carfluzepate	417.8	118.3	2.9	7	1	1	0	7.2
Doxefazepam	348.8	92.7	2.0	5	2	1	0	7.2
Cinolazepam	357.8	57.1	2.9	5	1	1	0	7.2
FG-8205	357.8	62.2	3.6	7	0	1	0	7.2
Ro 17-1812	357.8	63.0	3.1	6	0	1	0	7.1
Ro 15-4941	345.8	63.0	3.1	6	0	1	0	7.1
Fluloprazolam	448.5	90.4	2.4	9	0	1	0	7.1
JQ1	457.0	36.5	5.6	6	0	1	1	7.1
PWZ-029	291.7	34.7	2.8	5	0	1	0	7.0
Arfendazam	344.8	61.8	4.4	5	0	1	0	7.0
Flupyrazapon/ Zolazepam	286.3	64.4	2.0	5	0	1	0	7.0
Sulazepam	300.8	5.7	4.0	2	0	1	0	7.0
Mexazolam	363.2	34.7	4.2	4	1	1	0	7.0
Premazepam	253.3	58.2	2.5	4	1	1	0	7.0
Ripazepam	268.3	58.2	2.3	5	1	1	0	7.0
Tolufazepam	487.4	126.8	5.0	5	0	1	0	6.9

7-Aminoflunitrazepam	283.3	64.4	2.2	4	2	1	0	6.9
Elfazepam	408.9	152.3	3.1	5	0	1	0	6.8
Clazolam	312.8	26.5	3.4	3	0	1	0	6.8
Fosazepam	360.8	151.9	3.0	4	0	1	0	6.7
Pivoxazepam	370.8	81.6	4.0	5	1	1	0	6.4
Triflubazam	334.3	62.1	4.0	4	0	1	0	6.4
Gidazepam	387.2	79.0	1.6	6	3	1	0	6.3
Ro 48-8684	411.5	65.2	4.4	7	0	1	0	6.3
Ro 48-6791	412.5	74.2	3.8	8	0	1	0	6.3
Flutazolam	376.8	76.6	2.7	5	1	1	0	6.3
Zomebazam	284.3	53.0	2.1	6	0	1	0	5.9
Carburazepam	329.8	57.5	2.9	5	2	1	0	5.9
