# Supplementary material for: The Psychonauts’ Benzodiazepines; Quantitative Structure-Activity Relationship (QSAR) Analysis and Docking Prediction of Their Biological Activity
Source: Pharmaceuticals (Basel). 2021 Jul 26;14(8):720. doi: 10.3390/ph14080720 (PMC8398354; doi:10.3390/ph14080720)
Supplement: Supplementary file 1 [file pharmaceuticals-14-00720-s001.zip › pharmaceuticals-1312155-supplementary/Table S5.pdf]

Table S5. Predicted value of biological activity (log1/c) for the 101 DBZDs identified by NPSfinder®. Log1/c represents the logarithm of the reciprocal of the molar inhibitory concentration (IC50)(nM) required to displace 50% of [3H]-diazepam from rat cerebral cortex.

Note: The molecules are listed in decreasing order of predicted log1/c. The higher log1/c values should correspond to a higher biological activity.

Molecule	SMILES	Predicted log1/c
**High predicted biological activity**		
Ro 09-9212	Clc1c(C2=NCC(=O)Nc3sc(Cl)cc23)cccc1	9.40
Ro 07-5193	Clc1c(c(F)ccc1)C1=NCC(=O)Nc2c1cc(Cl)cc2	9.06
Ro 20-8065	Clc1c(Cl)cc2NC(=O)CN=C(c3c(F)cccc3)c2c1	9.04
Ro 07-5220	Clc1c(c(Cl)ccc1)C1=NCC(=O)N(C)c2c1cc(Cl)cc2	8.95
Ro 07-3953	Clc1cc2C(c3c(F)cccc3F)=NCC(=O)Nc2cc1	8.81
Flucotizolam	Clc1sc2-n3c(C)nnc3CN=C(c3c(F)cccc3)c2c1	8.77
Ciclotizolam	Brc1sc2-n3c(C4CCCCC4)nnc3CN=C(c3c(Cl)cccc3)c2c1	8.77
Flubrotizolam	Brc1sc2-n3c(C)nnc3CN=C(c3c(F)cccc3)c2c1	8.67
Phenazepam	Brc1cc2C(c3c(Cl)cccc3)=NCC(=O)Nc2cc1	8.61
Ro 07-9749	Ic1cc2C(c3c(F)cccc3)=NCC(=O)Nc2cc1	8.60
Clonazolam	Clc1c(C2=NCc3n(c(C)nn3)-c3c2cc([N+](=O)[O-])cc3)cccc1	8.58
Ro 15-9270	Clc1c(C=2c3c(-n4c(C)nnc4CC=2)ccc([N+](=O)[O-])c3)cccc1	8.52
Climazolam	Clc1c(C2=NCc3n(c(C)nc3)-c3c2cc(Cl)cc3)cccc1	8.49
Flunitrazolam	Fc1c(C2=NCc3n(c(C)nn3)-c3c2cc([N+](=O)[O-])cc3)cccc1	8.47
Ro 20-8552	Clc1c(C)cc2C(c3c(F)cccc3)=NCC(=O)Nc2c1	8.42
Methyl Clonazepam	Clc1c(C2=NCC(=O)N(C)c3c2cc([N+](=O)[O-])cc3)cccc1	8.40
Reclazepam	Clc1c(C2=NCCN(C=3OCC(=O)N=3)c3c2cc(Cl)cc3)cccc1	8.39
Uldazepam	Clc1c(C2=NCC(NOCC=C)=Nc3c2cc(Cl)cc3)cccc1	8.39
Zapizolam	Clc1c(C2=NCc3n(-c4c2nc(Cl)cc4)cnn3)cccc1	8.38
Ethyl Dirazepate	Clc1c(C2=NC(C(=O)OCC)C(=O)Nc3c2cc(Cl)cc3)cccc1	8.35
Difludiazepam (RO- 07-4065)	Clc1cc2C(c3c(F)cccc3F)=NCC(=O)N(C)c2cc1	8.35
Metizolam	Clc1c(C2=NCc3n(-c4sc(CC)cc24)cnn3)cccc1	8.35
Etizolam	Clc1c(C2=NCc3n(c(C)nn3)-c3sc(CC)cc23)cccc1	8.34
Desmethyltriazolam	Clc1c(C2=NCc3n(-c4c2cc(Cl)cc4)cnn3)cccc1	8.32
Flubromazepam	Brc1cc2C(c3c(F)cccc3)=NCC(=O)Nc2cc1	8.30
Ro 13-3780	Brc1cc2C(c3c(F)cccc3F)=NCC(=O)N(C)c2cc1	8.26
Lopirazepam	Clc1c(C2=NC(O)C(=O)Nc3c2nc(Cl)cc3)cccc1	8.24
Cloniprazepam	Clc1c(C2=NCC(=O)N(CC3CC3)c3c2cc([N+](=O)[O-])cc3)cccc1	8.23
Nifoxipam (3-OH-Norflunitrazepam)	Fc1c(C2=NC(O)C(=O)Nc3c2cc([N+](=O)[O-])cc3)cccc1	8.21
Phenazolam (Clobromazolam, DM-ii-90)	Brc1cc2C(c3c(Cl)cccc3)=NCc3n(c(C)nn3)-c2cc1	8.20
Diclazepam	Clc1c(C2=NCC(=O)N(C)c3c2cc(Cl)cc3)cccc1	8.19
4'-Chlorodiazepam	Clc1cc2C(c3ccc(Cl)cc3)=NCC(=O)N(C)c2cc1	8.19
3-Hydroxyphenazepam	Brc1cc2C(c3c(Cl)cccc3)=NC(O)C(=O)Nc2cc1	8.16
Bentazepam	O=C1Nc2sc3c(c2C(c2ccccc2)=NC1)CCCC3	8.16

Cinazepam	Brc1cc2C(c3c(Cl)cccc3)=NC(OC(=O)CCC(=O)O)C(=O)Nc2cc1	8.16
Flualprazolam	Clc1cc2C(c3c(F)cccc3)=NCc3n(c(C)nn3)-c2cc1	8.15
Metaclazepam	Brc1cc2C(c3c(Cl)cccc3)=NCC(COC)N(C)c2cc1	8.14
Fluetizolam	Fc1c(C2=NCc3n(c(C)nn3)-c3sc(CC)cc23)cccc1	8.14
Pynazolam	O=[N+]([O-])c1cc2C(c3ncccc3)=NCc3n(c(C)nn3)-c2cc1	8.13
Desmethylnitrazolam	O=[N+]([O-])c1cc2C(c3ccccc3)=NCc3n(-c2cc1)cnn3	8.11
Nitrazolam	O=[N+]([O-])c1cc2C(c3ccccc3)=NCc3n(c(C)nn3)-c2cc1	8.07
Flubromazolam	Brc1cc2C(c3c(F)cccc3)=NCc3n(c(C)nn3)-c2cc1	8.00
**Medium predicted biological activity**		
Fletazepam	Clc1cc2C(c3c(F)cccc3)=NCC(=O)N(CC(F)(F)F)c2cc1	7.99
7-BPDBD	Brc1cc2C(c3ccccc3)=NCC(=O)Nc2cc1	7.90
Tuclazepam	Clc1c(C2=NCC(CO)N(C)c3c2cc(Cl)cc3)cccc1	7.88
SH-053-R-CH3-2'F	Fc1c(C2=NC(C)c3c(C(=O)OCC)ncn3-c3c2cc(C#C)cc3)cccc1	7.81
Pyclazolam	Clc1cc2C(c3ncccc3)=NCc3n(c(C)nn3)-c2cc1	7.80
RO 21-8137	Clc1cc2C(c3c(F)cccc3)=NCc3c(C(=O)N)ncn3-c2cc1	7.79
Estazolam	Clc1cc2C(c3ccccc3)=NCc3n(-c2cc1)cnn3	7.78
Pyeazolam (SH-TRI-108)	C(#C)c1cc2C(c3ncccc3)=NCc3n(c(C)nn3)-c2cc1	7.78
Flutoprazepam	Clc1cc2C(c3c(F)cccc3)=NCC(=O)N(CC3CC3)c2cc1	7.77
Rilmazolam	Clc1c(C2=NCc3n(nc(C(=O)N(C)C)n3)-c3c2cc(Cl)cc3)cccc1	7.77
Deschloroetizolam	C(C)c1sc2-n3c(C)nnc3CN=C(c3ccccc3)c2c1	7.73
Zometapine	Clc1cc(C2=NCCNc3n(C)nc(C)c23)ccc1	7.65
Pypazolam	Brc1cc2C(c3ncccc3)=NCc3n(c(C)nn3)-c2cc1	7.64
Imidazenil	Brc1c(C2=NCc3c(C(=O)N)ncn3-c3c2cc(F)cc3)cccc1	7.64
Menitrazepam	O=[N+]([O-])c1cc2C(C3=CCCCC3)=NCC(=O)N(C)c2cc1	7.60
Bromazolam	Brc1cc2C(c3ccccc3)=NCc3n(c(C)nn3)-c2cc1	7.59
MP-iii-022	Fc1c(C2=NC(C)c3c(C(=O)NC)ncn3-c3c2cc(C#C)cc3)cccc1	7.58
Ro 05-4608	Clc1c(C2=NCC(=O)N(C)c3c2cccc3)cccc1	7.55
Cyprazepam	Clc1cc2C(=[N+]([O-])CC(NCC3CC3)=Nc2cc1)c1ccccc1	7.53
Triflunordazepam	FC(F)(F)c1cc2C(c3ccccc3)=NCC(=O)Nc2cc1	7.52
Quazepam	Clc1cc2C(c3c(F)cccc3)=NCC(=S)N(CC(F)(F)F)c2cc1	7.51
N-Methylbromazepam	Brc1cc2C(c3ncccc3)=NCC(=O)N(C)c2cc1	7.48
Flutemazepam	Clc1cc2C(c3c(F)cccc3)=NC(O)C(=O)N(C)c2cc1	7.48
Thionordazepam	Clc1cc2C(c3ccccc3)=NCC(=S)Nc2cc1	7.45
Iomazenil	Ic1c2C(=O)N(C)Cc3c(C(=O)OCC)ncn3-c2ccc1	7.44
QH-II-066	O=C1N(C)c2c(C(c3ccccc3)=NC1)cc(C#C)cc2	7.42
CP-1414S	O=[N+]([O-])c1cc2N(c3ccccc3)C(=O)CC(N)=Nc2cc1	7.42
Lofendazam	Clc1cc2N(c3ccccc3)C(=O)CCNc2cc1	7.41
Tofisopam	O(C)c1c(OC)ccc(C2=NN=C(C)C(CC)c3c2cc(OC)c(OC)c3)c1	7.36
Fluadinazolam	Clc1cc2C(c3c(F)cccc3)=NCc3n(c(CN(C)C)nn3)-c2cc1	7.33
Remimazolam	Brc1cc2C(c3ncccc3)=NC(CCC(=O)OC)c3n(c(C)cn3)-c2cc1	7.32
Ethyl Carfluzepate	Clc1cc2C(c3c(F)cccc3)=NC(C(=O)OCC)C(=O)N(C(=O)NC)c2cc1	7.24
Doxefazepam	Clc1cc2C(c3c(F)cccc3)=NC(O)C(=O)N(CCO)c2cc1	7.23

Cinolazepam	Clc1cc2C(c3c(F)cccc3)=NC(O)C(=O)N(CCC#N)c2cc1	7.20
FG-8205	Clc1c2C(=O)N(C)Cc3c(-c4nc(C(C)C)on4)ncn3-c2ccc1	7.17
Ro 17-1812	Clc1c2C(=O)N3C(c4c(C(=O)OCC5CC5)ncn4-c2ccc1)CC3	7.13
Ro 15-4941	Clc1c2C(=O)N3C(c4c(C(=O)OCC)ncn4-c2ccc1)CCC3	7.09
Fluloprazolam	Fc1c(C2=NCC=3N(C(=O)C(=CN4CCN(C)CC4)N=3)c3c2cc([N+](=O)[O-])cc3)cccc1	7.08
JQ1	Clc1ccc(C2=NC(CC(=O)OC(C)(C)C)c3n(c(C)nn3)-c3sc(C)c(C)c23)cc1	7.06
PWZ-029	Clc1cc2C(=O)N(C)Cc3c(COC)ncn3-c2cc1	7.04
Arfendazam	Clc1cc2N(c3ccccc3)C(=O)CCN(C(=O)OCC)c2cc1	7.03
Flupyrazapon/ Zolazepam	Fc1c(C2=NCC(=O)N(C)c3n(C)nc(C)c23)cccc1	7.00
Sulazepam	Clc1cc2C(c3ccccc3)=NCC(=S)N(C)c2cc1	7.00
**Low predicted biological activity**		
Mexazolam	Clc1c(C23OCC(C)N2CC(=O)Nc2c3cc(Cl)cc2)cccc1	6.98
Premazepam	O=C1Nc2c(c(C)n(C)c2)C(c2ccccc2)=NC1	6.97
Ripazepam	O=C1Nc2c(C)nn(CC)c2C(c2ccccc2)=NC1	6.96
Tolufazepam	Clc1c(C2=NCC(=O)N(CCS(=O)(=O)c3ccc(C)cc3)c3c2cc(Cl)cc3)cccc1	6.95
7-Aminoflunitrazepam	Fc1c(C2=NCC(=O)N(C)c3c2cc(N)cc3)cccc1	6.95
Elfazepam	Clc1cc2C(c3c(F)cccc3)=NCC(=O)N(CCS(=O)(=O)CC)c2cc1	6.85
Clazolam	Clc1cc2c(N(C)C(=O)CN3C2c2c(cccc2)CC3)cc1	6.78
Fosazepam	Clc1cc2C(c3ccccc3)=NCC(=O)N(CP(=O)(C)C)c2cc1	6.66
Pivoxazepam	Clc1cc2C(c3ccccc3)=NC(OC(=O)C(C)(C)C)C(=O)Nc2cc1	6.45
Triflubazam	FC(F)(F)c1cc2N(c3ccccc3)C(=O)CC(=O)N(C)c2cc1	6.43
Gidazepam	Brc1cc2C(c3ccccc3)=NCC(=O)N(CC(=O)NN)c2cc1	6.34
Ro 48-8684	Fc1cc2C(=O)N(C)Cc3c(-c4oc(CN(CCC)CCC)cn4)ncn3-c2cc1	6.33
Ro 48-6791	Fc1cc2C(=O)N(C)Cc3c(-c4nc(CN(CCC)CCC)on4)ncn3-c2cc1	6.29
Flutazolam	Clc1cc2C3(c4c(F)cccc4)OCCN3CC(=O)N(CCO)c2cc1	6.26
Zomebazam	O=C1N(C)c2n(C)nc(C)c2N(c2ccccc2)C(=O)C1	5.95
Carburazepam	Clc1cc2C(N(C(=O)N)CC(=O)N(C)c2cc1)c1ccccc1	5.86
